# The Fitness Consequences of Aneuploidy Are Driven by Condition-Dependent Gene Effects

**DOI:** 10.1371/journal.pbio.1002155

**Published:** 2015-05-26

**Authors:** Anna B. Sunshine, Celia Payen, Giang T. Ong, Ivan Liachko, Kean Ming Tan, Maitreya J. Dunham

**Affiliations:** 1 Department of Genome Sciences, University of Washington, Seattle, Washington, United States of America; 2 Department of Biostatistics, University of Washington, Seattle, Washington, United States of America; Institute of Science and Technology Austria (IST Austria), AUSTRIA

## Abstract

Aneuploidy is a hallmark of tumor cells, and yet the precise relationship between aneuploidy and a cell’s proliferative ability, or cellular fitness, has remained elusive. In this study, we have combined a detailed analysis of aneuploid clones isolated from laboratory-evolved populations of *Saccharomyces cerevisiae* with a systematic, genome-wide screen for the fitness effects of telomeric amplifications to address the relationship between aneuploidy and cellular fitness. We found that aneuploid clones rise to high population frequencies in nutrient-limited evolution experiments and show increased fitness relative to wild type. Direct competition experiments confirmed that three out of four aneuploid events isolated from evolved populations were themselves sufficient to improve fitness. To expand the scope beyond this small number of exemplars, we created a genome-wide collection of >1,800 diploid yeast strains, each containing a different telomeric amplicon (Tamp), ranging in size from 0.4 to 1,000 kb. Using pooled competition experiments in nutrient-limited chemostats followed by high-throughput sequencing of strain-identifying barcodes, we determined the fitness effects of these >1,800 Tamps under three different conditions. Our data revealed that the fitness landscape explored by telomeric amplifications is much broader than that explored by single-gene amplifications. As also observed in the evolved clones, we found the fitness effects of most Tamps to be condition specific, with a minority showing common effects in all three conditions. By integrating our data with previous work that examined the fitness effects of single-gene amplifications genome-wide, we found that a small number of genes within each Tamp are centrally responsible for each Tamp’s fitness effects. Our genome-wide Tamp screen confirmed that telomeric amplifications identified in laboratory-evolved populations generally increased fitness. Our results show that Tamps are mutations that produce large, typically condition-dependent changes in fitness that are important drivers of increased fitness in asexually evolving populations.

## Abstract 2

A novel method to create thousands of aneuploid strains in *Saccharomyces cerevisiae* allows the authors to dissect the complex role of aneuploidy in adaptation and evolution.

## Author Summary

Aneuploidy (altered copy number of genomic regions) is observed in the majority of tumors, but it remains unclear whether aneuploidy is a cause or consequence of cancer. Evidence from the yeast *Saccharomyces cerevisiae* and mammalian cells has shown that aneuploid cells tend to grow more slowly than normal cells; however, aneuploidy has also been shown to promote tumor formation and microbial adaptation. To address this paradox, we took two approaches to study the relationship between fitness—measured as cellular growth—and aneuploidy. First, we examined aneuploid events isolated from laboratory-evolved populations of *S*. *cerevisiae* and found that the majority of such events improve cellular fitness, have a large effect-size, and show diverse fitness effects under different conditions. Second, we developed a method to create thousands of aneuploid strains spanning the yeast genome and used pooled competition experiments followed by barcode sequencing to determine their relative fitnesses. These genome-wide data revealed aneuploidy to have effects that were both large and wide-ranging (pleiotropic). We found that both the positive and negative fitness effects are typically driven by a small number of genes within each aneuploidy event. We conclude that aneuploidy is functionally important in the process of adaptation of yeast during laboratory evolution experiments and propose that it has the potential to play an adaptive role during the evolution of cancers.

## Introduction

Aneuploidy, a class of mutation infamous for its disruption of development [1] and oncogenic connections [2,3], is a genetic alteration that changes the copy number of many genes with a single mutational event (reviewed in [4]). Despite its close connection to cancer, a phenomenon characterized by unchecked cellular proliferation, aneuploidy has been shown to inhibit cellular growth in a variety of model systems. Both trisomic mouse embryonic fibroblasts and disomic strains of *Saccharomyces cerevisiae* have increased doubling times when compared to their euploid counterparts [3,5]. The fitness cost associated with aneuploidy has been attributed to proteotoxic stress caused by the unbalanced and uncompensated expression of proteins from the regions of altered copy number [6–9].

Despite this general fitness cost, whole-chromosomal aneuploidy and segmental aneusomy, both of which will henceforth be referred to as “aneuploidy” for simplicity, have been commonly observed in the evolution and adaptation of asexually replicating cells [10–20]. Aneuploidy thus has a paradoxical relationship with cellular fitness [21]: while typically decreasing a cell’s fitness, it is nonetheless selected for under a variety of highly selective conditions. By altering the copy number of multiple genes at once, it has been argued that aneuploidy allows a cell to explore a wide fitness landscape [22,23]. Aneuploidy, therefore, may commonly be selected for when cells face novel conditions because this mutation type allows an evolving population to rapidly test many divergent phenotypes. However, the specific fitness effects of aneuploid events have been difficult to directly test and, instead, have typically been inferred from their recurrence between or frequency within evolving populations [13,14,24]. Even in the rare cases in which a fitness advantage is directly associated with a particular aneuploid event, it remains challenging to identify the gene(s) within the aneuploid region whose altered copy number is responsible for the fitness effects observed [25,26]. However, the gene(s) underlying the phenotype(s) associated with an aneuploid event have been identified in a small number of cases [17,20,27].

Aneuploidy’s genetic complexity and the challenges outlined above have made it difficult to draw firm conclusions about the general role aneuploidy plays in fitness, adaptation, and evolution. In this study, we have directly tested the fitness effects of four naturally selected aneuploid events isolated from three laboratory evolution experiments of *S*. *cerevisiae* carried out in nutrient-limited chemostats. We have found that while most aneuploid events positively affect fitness, one event actually decreased fitness despite representing a substantial fraction of the population. Unable to draw general conclusions about aneuploidy from the detailed analysis of only a few specific genetically tractable events, we then created a barcoded genomic collection of >1,800 clones each containing a telomeric amplification (Tamp) of a different size. By tiling across the entire yeast genome, this collection allowed us to test the fitness effects of telomeric amplifications genome-wide. Using pooled competition experiments in glucose-, sulfate-, or phosphate-limited chemostats combined with barcode sequencing [28], we have uncovered the fitness profile explored by Tamps under these three conditions. Data from this genome-wide Tamp screen revealed that aneuploidy is typically a large-effect mutation, with condition-specific fitness effects and fitness tradeoffs under alternative conditions. By comparing the Tamp screen data to aneuploid events identified in evolution experiments, we found that most aneuploid events identified in evolution experiments positively affect fitness. The discrete fitness breakpoints in the Tamp fitness profile allowed us to identify candidate driver genes that, in the genetic background of amplification of contiguous genes, were responsible for the fitness effects of each Tamp. We discovered that the fitness effects of most aneuploid events from evolved populations are driven by a small number of driver genes essential for their positive effects on competitive growth. These data are an attempt to systematically define the fitness landscape explored by aneuploidy.

## Results

### Aneuploid Events Rise to High Population Frequencies in Laboratory Evolution Experiments

Aneuploidy has been commonly observed in laboratory-evolved populations of *S*. *cerevisiae* adapting to nutrient limitation [10,12,29,30]. Our group previously reported that at least one aneuploid clone was observed in 13 out of 24 evolution experiments carried out for over 100 generations (122–328 generations) in nutrient-limiting chemostats [10]. The same study showed that all eight of the evolution experiments carried out under sulfate-limiting conditions contained a recurrent amplification surrounding the high-affinity sulfate transporter *SUL1* [10]; two sulfate-limited populations contained aneuploid events in addition to the *SUL1* amplification. The direct fitness effects and mechanism of formation regarding the *SUL1* amplicon have been examined in detail elsewhere [30–32]; in this study our primary focus was to explore the functional importance of the remaining aneuploid events observed in the 24 evolution experiments.

**Fig 1:**
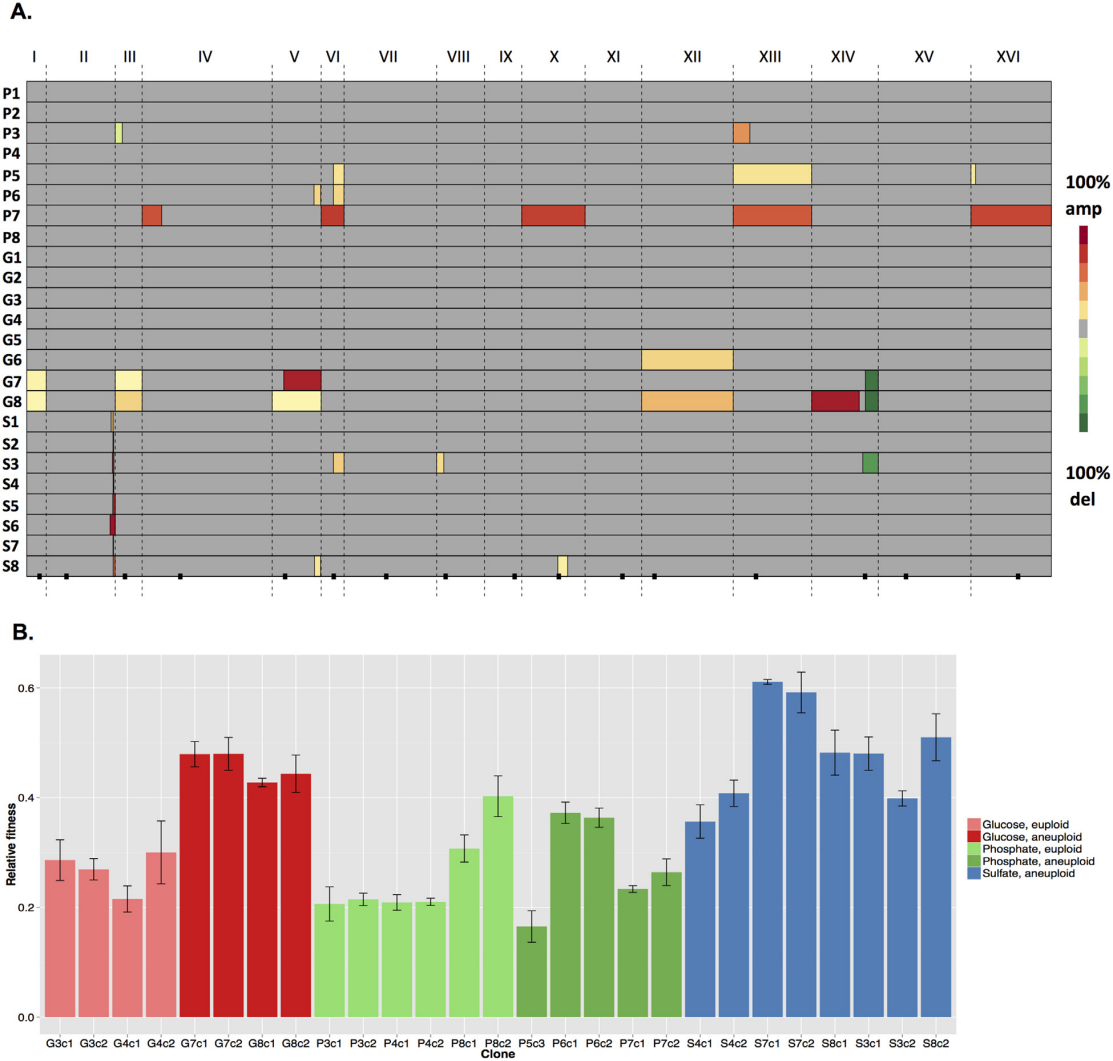
Aneuploid events rise to high population frequencies in evolution experiments, and aneuploid clones are more fit than their euploid, wild-type ancestor. A) Each row represents an independent evolution experiment and is named according to [10], where “P” indicates a phosphate-limited evolution experiment, “S” indicates a sulfate-limited evolution experiment, and “G” indicates a glucose-limited evolution experiment. The dotted vertical lines distinguish the 16 yeast chromosomes and the black squares mark the position of each centromere. Aneuploid events are defined as red or green boxes where shades of red indicate amplifications and shades of green boxes indicate deletions. The population frequency of each aneuploid event is represented as a color along the red-green color gradient shown to the right. Grey regions indicate euploid regions. Raw data can be found in S1 Table. B) The relative fitness of evolved clones isolated from evolution experiments carried out under glucose-limited (red), phosphate-limited (green), or sulfate-limited conditions (blue) are shown as the mean +/- standard error (SE). Aneuploid clones isolated from glucose-limited evolution experiments show a significantly greater fitness than euploid clones (Wilcoxon Rank Sum test, *p*-value = 0.029). Clones isolated from sulfate-limited evolution experiments show significantly greater fitness than the glucose-limited or phosphate-limited evolved clones (Wilcoxon Rank-Sun test, *p*-value = 0.036). Raw data can be found in S2 Table.

As a proxy for their direct fitness effects, we first determined the population frequencies of the aneuploid events observed in the 24 evolution experiments [10] using array comparative genomic hybridization (aCGH) of population DNA. We predicted that aneuploid events rising to appreciable population frequencies provided a fitness advantage to the clones carrying them. In the initial description of the evolution experiments examined here, population aCGH was performed on 10 of the 24 evolution experiments [10]; here we performed population aCGH on the remaining 14 evolution experiments (Fig 1A and S1 Table, see GEO Accession GSE67769 for raw data). Given the tandem repeat structure of the *SUL1* amplicons [30,32], their clonal copy number was dynamic and prohibited accurate calculation of their population frequency by population aCGH. In order to estimate the *SUL1* amplicon population frequency, we defined the *SUL1* clonal copy number as the population copy number rounded to the next highest integer. A detailed analysis of the *SUL1* amplicon structure and population dynamics has been presented elsewhere [30].

The aneuploid events present in the evolution experiments ranged in size from 5–1,000 kb and were present at frequencies ranging from 6% (our lower limit of detection) to 96% of the population with an average population frequency of 47% (Fig 1A). We confirmed the accuracy of this approach by performing breakpoint PCR across the translocation event in the supernumerary chromosome present in population S8. Both aCGH on S8 population DNA and breakpoint PCR on 98 independent clones isolated from S8 determined this supernumerary chromosome to be present at 13% of the population (see GEO Accession GSE67769).

While 11 of the aneuploid events were unique, seven recurred between populations, both within and between conditions, most notably the amplification on the right arm of chromosome V and the amplification on the left arm of chromosome XIV. The amplification on the right arm of chromosome V recurred in three different evolution experiments carried out under three conditions (Fig 1A), while the amplification of the left arm of chromosome XIV was observed in one of the glucose-limited evolution experiments described here and in two additional glucose-limited evolution experiments previously analyzed [12]. The high population frequencies and the recurrence of aneuploid events between populations supported our hypothesis that the aneuploid events examined here were selected for under the conditions of laboratory evolution.

### Evolved Clones Isolated from Laboratory Evolution Experiments Have Increased Fitness

We next asked whether aneuploid and euploid evolved clones isolated from the final generation of evolution experiments were more fit than their wild-type ancestors. We determined the relative fitness of each evolved clone through chemostat competition experiments against an appropriate green fluorescent protein (GFP)-marked wild-type control clone and under conditions identical to the evolution experiment from which the evolved clone was isolated. Both euploid and aneuploid evolved clones showed a fitness advantage relative to their wild-type ancestor (Fig 1B and S2 Table). Note that clones P3c1 and P3c2 are euploid despite being isolated from an aneuploid population, because the aneuploid events were not fixed in population P3. The relative fitnesses of the evolved clones ranged from 17% to 61% more fit than the wild-type ancestor. Evolved clones isolated from sulfate-limited evolution experiments (*n* = 8) had significantly greater fitnesses than clones isolated from glucose or phosphate-limited evolution experiments (*n* = 19) (Wilcoxon Rank-Sum test, *p*-value = 0.036). While there was a statistical difference in the relative fitnesses between euploid and aneuploid clones (Wilcoxon Rank-Sum test, *p*-value = 0.0014), this was driven in part by the high fitness conferred by the *SUL1* amplicon in all evolved clones isolated from sulfate-limited evolution experiments. However, the relationship between aneuploidy and fitness held true even when we restricted our examination to the eight clones isolated under glucose-limiting conditions: aneuploid clones (*n* = 4) had a significantly greater fitness than the euploid clones (*n* = 4) (Wilcoxon Rank Sum test, *p*-value = 0.029). Although these data demonstrated that evolved aneuploid clones, just like evolved euploid clones, are more fit than their wild-type ancestor, it did not establish whether the aneuploid events themselves or other mutations, such as single-nucleotide variants (SNVs), contributed to the improved fitness of evolved clones. To provide this direct connection we genetically isolated the aneuploid events and SNVs from three evolved aneuploid clones and determined the direct fitness consequences of both the aneuploid events and the SNVs.

### Aneuploid Events Increased the Fitness of Evolved Clones

To genetically isolate the aneuploid events present in evolved clones we first determined the full repertoire of mutations present in a subset of evolved clones. We chose to study three evolved aneuploid clones: two clones isolated at generations 141 and 217 (P5c3 and P6c1) from phosphate-limited evolution experiments begun with a haploid founder and one clone isolated at generation 250 (S8c2) from a sulfate-limited evolution experiment begun with a diploid founder. P5c3 has two aneuploid events: an extra copy of chromosome XIII and a supernumerary chromosome consisting of the right arm of chromosome VI joined to a telomeric amplicon from the left side of chromosome XVI (VI_R_ t XVI_L_). P6c1 has a supernumerary chromosome consisting of a telomeric amplicon on the right side of chromosome V joined to the right arm of chromosome VI (V_R_ t VI_R_) and S8c2 contains a supernumerary chromosome consisting of two copies of a telomeric amplicon from the right side of chromosome V flanking a centromeric segmental amplicon from chromosome X (V_R_ t X_CEN_). These clones were chosen because they did not contain large deletions, thus making them amenable to backcrossing and tetrad dissection. We performed whole-genome sequencing (WGS) of these clones to an average mapping coverage of 46–68X (S3 Table). Three to seven SNVs were called in each clone (Table 1) and confirmed by Sanger sequencing. We also sequenced the populations from which clones P5c3 and P6c1 were isolated to an average mapping coverage of 39X and 116X, respectively, and determined that the SNVs identified in these clones ranged in frequency from below detection to 98% (Table 1).

**Table 1:** Point mutations in evolved clones identified by WGS.

Clone	Position	Mutation	Gene	AA change	Population Frequency	Recurrent?	Fitness (mean ± SE)
P5c1	chrII: 720006	G->T	*SPO23*	S325I	0.05		0.04 ± 0.009
	chrXIII: 136355	G-> C	*POB3*	V286L	0		0.006 ± 0.007
	chrXIII: 885153	T-> C	*FSK3*	F1332S	0		0.051 ± 0.012
	VI_R_ t XVI_L_ chr				0.17	Y (VI_R_)	0.159 ± 0.009
	chrXIII disomy				0.17		0.15 ± 0.024
P6c1	chrXVI: 619455	G-> T	intergenic		1		ND
	chrIV: 1408563	G-> A	*JIP4*	A511V	0		0.003 ± 0.003
	chrV: 545020	C-> A	*ECM32*	Q1111K	0.14		-0.012 ± 0.002
	chrVIII: 401803	G-> C	*SPO12*	V124L	1		0.048 ± 0.022
	chrXIII: 25655	G-> A	*PHO84*	A49V	0.98	Y	0.233 ± 0.013
	chrXIII: 499172	C-> T	*MGR3*	T490I	0		0.015 ± 0.01
	chrXIV: 381852	G-> A	*NRK1*	V125I	0		0.027 ± 0.007
	V_R_ t VI_R_ chr				0.23	Y	0.113 ± 0.011
S8c2	chrXIV: 299460	C-> A	*YNL181W*	Q376K	ND		-0.061 ± 0.008
	chrIV: 47386	C-> G	*HO*	L216V	ND		-0.031 ± 0.018
	chrIV: 897313	G-> A	*ADR1*	S761N	ND		-0.119 ± 0.023
	*SUL1* amp.				ND	Y	0.533 ± 0.012
	V_R_ t X_CEN_ chr				0.13	Y (V_R_)	-0.10 ± 0.016

**Fig 2:**
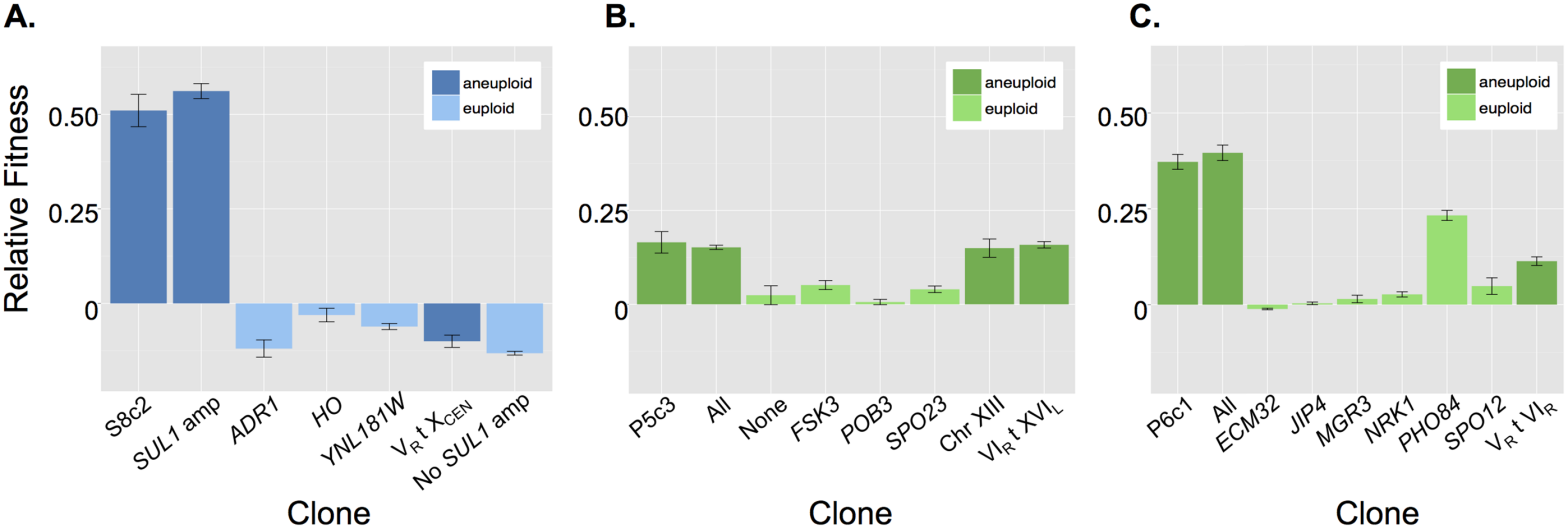
Aneuploidy variably affects fitness of evolved clones. The individual fitness effects of point mutations and aneuploid events were determined for all mutations identified in the evolved clones S8c2 (A), P5c3 (B), and P6c1 (C). The supernumerary chromosome(s) in each clone are labeled by their identifying translocation or, in the case of the chromosome XIII disomy, as “Chr XIII.” Aneuploid and euploid clones are color-coded according to the legend. As described in the text, “All” for P5c3 and P6c1 and “None” for P5c3 represent backcrossed segregants that contain all or none of the mutations present in the original evolved clone. “No *SUL1* amp” for S8c2 is a backcrossed segregant that contains all the mutations identified in S8c2 except for the *SUL1* amplicon. Raw data can be found in S2 Table.

To isolate segregants that had a single evolved SNV or aneuploid event in an otherwise ancestral genetic background, we backcrossed the haploid clones P5c3 and P6c1 to their corresponding wild-type strain and directly sporulated the diploid clone S8c2. We identified appropriate segregants by genotyping and then used chemostat competition experiments to determine the independent fitness effects of each evolved mutation (Fig 2 and S2 Table). More than half of the mutations examined showed either neutral/near-neutral (<5%) fitness increase or negative effects on fitness, agreeing with previous reports that genetic hitchhiking is quite important for the spectrum of mutations observed in asexually evolving populations [33,34]. In particular, the supernumerary chromosome isolated from evolved clone S8c2, despite occupying 13% of the S8 population, actually decreased the fitness of clones carrying it by 10% (Fig 2). A minority of evolved mutations, including three large-scale aneuploid events, the amplification of *SUL1*, and a missense mutation in the high-affinity phosphate transporter *PHO84*, all increased fitness in the conditions from which they were isolated.

In general, the aneuploid events we examined showed diverse relationships with the overall fitnesses of the evolved clones from which they were isolated. In P6c1, the fitness effect of the V_R_ t VI_R_ supernumerary chromosome added to a second positive-effect mutation, the missense mutation in *PHO84*, to predict the overall fitness of the original evolved clone. In contrast, in P5c3 the positive fitnesses associated with both aneuploid events in that clone were each similar to the overall fitness of the original evolved clone, suggesting epistasis between these two mutations. Finally, the overall fitness of evolved clone S8c2 was quite similar to the fitness effect of the *SUL1* amplification alone, suggesting epistasis between the *SUL1* amplicon and the 10% fitness cost conferred by the V_R_ t X_CEN_ supernumerary chromosome and the missense mutation in *ADR1* in this clone.

To confirm that we had identified and genetically isolated all functionally important mutations, for P5c3 and P6c1 we isolated and determined the relative fitness of backcrossed segregants that either had all or, in the case of P5c3 alone, none of the mutations present in the original evolved clone. As expected, the backcrossed segregants with all of the evolved mutations had a relative fitness similar to the original evolved clone, while the P5c3 backcrossed segregant with none of the evolved mutations had neutral fitness (Fig 2B and 2C). We were unable to isolate similar backcrossed clones corresponding to S8c2. However, given the negative fitness effects of the V_R_ t X_CEN_ supernumerary chromosome in S8c2, we were particularly interested to see if there was any epistasis, and specifically sign epistasis, between the point mutations and the V_R_ t X_CEN_ supernumerary chromosome in S8c2. To test this, we determined the relative fitness of a backcrossed clone with all of the evolved mutations except for the *SUL1* amplicon (“No *SUL1* amp” in Fig 2A). This clone had a fitness of -13% which, given the >5% fitness deficit of the V_R_ t X_CEN_ supernumerary chromosome, the *HO* mutation, and the *YNL181W* mutation, indicated epistasis between these mutations, although sign epistasis was not observed.

### Aneuploid Events Are Pleiotropic

When organisms adapt to a particular environment they may acquire mutations that produce a fitness tradeoff under alternative conditions [35,36]. Aneuploid events have previously been proposed to be pleiotropic mutations that, over the course of a population’s adaptation to a novel environment, are eventually replaced by mutations with fewer non-selective effects and correspondingly fewer fitness tradeoffs [37]. With these observations in mind, we determined the growth rates for 20 of the evolved clones in batch culture in rich media and observed a significant decrease in growth rate relative to wild-type for three of the 20 clones (S1 Fig)

**Fig 3:**
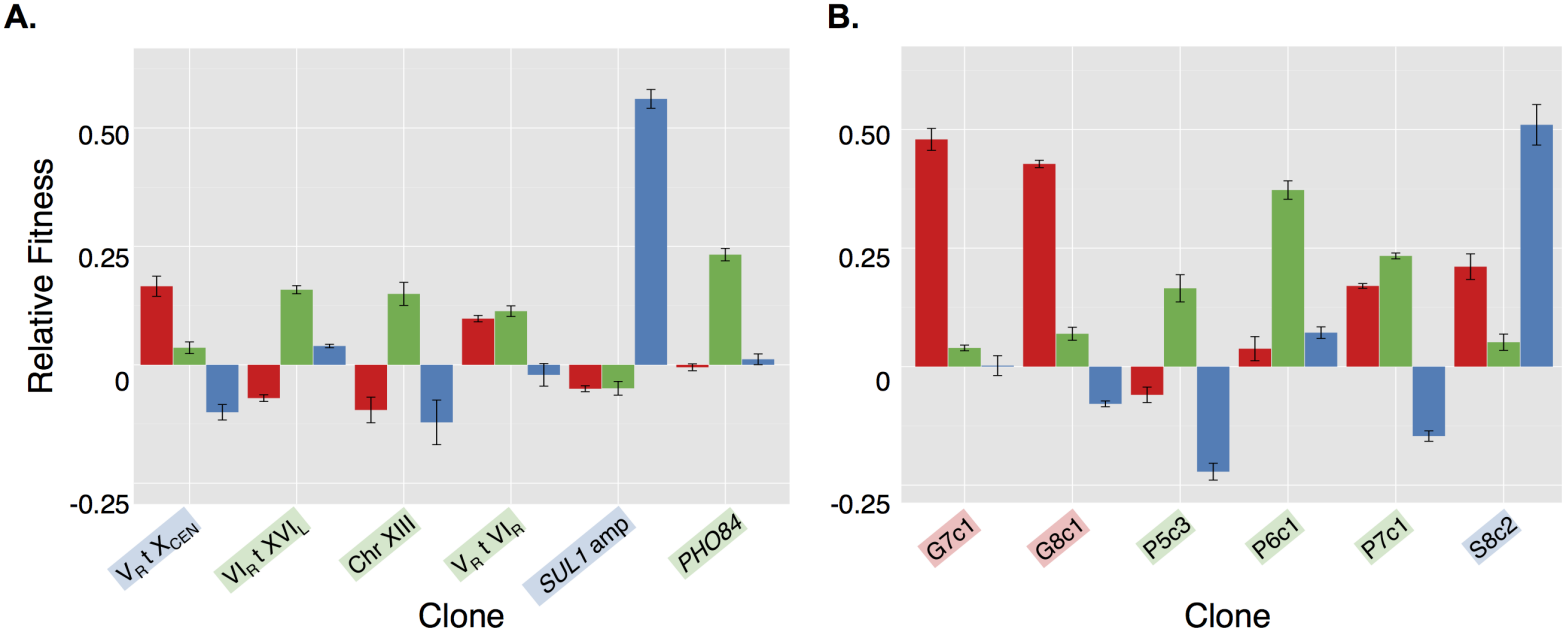
Aneuploid events variably affect fitness under alternative conditions. In both A) and B), the name of each aneuploid event or aneuploid clone, respectively, is highlighted with the color corresponding to the condition from which it was originally isolated, while the color of the bar indicates the conditions under which the relative fitness was determined: glucose- (red), sulfate- (blue) and phosphate-limitation (green). A) Aneuploid events identified in evolution experiments and isolated into an otherwise wild-type background show divergent fitness effects under the three conditions examined. The supernumerary chromosomes are labeled according to their identifying translocation (i.e., V_R_ t X_CEN_) and the chromosome XIII disomy is labeled as “Chr XIII.” The missense mutation in *PHO84* identified in the phosphate-limited evolution experiment P6 only affects fitness under phosphate-limiting conditions and has no effect on fitness under glucose- or sulfate-limiting conditions. B) Evolved aneuploid clones have divergent fitness effects under the three conditions examined here. G, P, and S indicate evolved clones isolated from glucose-, phosphate-, and sulfate-limited evolution experiments, respectively. Raw data can be found in S2 Table.

The similar doubling times to wild-type for most of the evolved clones suggested that the majority of evolved clones do not show a fitness tradeoff under typical lab growth conditions. However, comparing monoculture growth rates is an insensitive method to detect small fitness differences between clones. We therefore examined the fitnesses of six evolved aneuploid clones and the four aneuploid events we had previously isolated (Fig 2) using chemostat competition experiments under the two nutrient limitation conditions not previously examined. Each of the four isolated aneuploid events and the *SUL1* amplicon showed different fitness effects in the two alternative conditions (Fig 3A). Typically, each aneuploid event decreased or had a small effect (<5%) on fitness under the two alternative conditions tested.

However, both the V_R_ t X_CEN_ supernumerary chromosome from the sulfate-limited population S8 and the V_R_ t VI_R_ supernumerary chromosome from phosphate-limited population P6 increased fitness under glucose-limited conditions. We next tested evolved aneuploid clones under the two nutrient limitation conditions not previously examined and observed results similar to those achieved with the isolated aneuploid events. The evolved aneuploid clones typically had lower-than-wild-type fitness under the two alternative nutrient conditions, although occasionally they had increased fitness under alternative conditions (Fig 3B). Finally, we compared the pleiotropy, defined as the variance in fitness between conditions, of the four isolated aneuploid events to the pleiotropy of single-gene changes in copy number and found aneuploid events to be significantly more pleiotropic than single-gene changes in copy number (unpaired, two-tailed *t* test, *p =* 0.049, S2A Fig) These results generally supported previous hypotheses that proposed aneuploidy to be highly pleiotropic [7,37]; however, these results also emphasized that aneuploidy does not always lead to negative fitness tradeoffs but can also have unselected fitness benefits under alternative conditions.

These detailed analyses of evolved aneuploid clones isolated from laboratory evolution experiments demonstrated the varying impact aneuploidy could exert on cellular fitness and proved that aneuploidy can cause fitness improvements in experimental evolution under nutrient limitation. However, this type of rigorous analysis is not scalable, and the limited number of clones we have examined here precluded any conclusions about the general effects of aneuploidy on fitness, adaptation, and evolution. With the dual goals of (1) identifying which aneuploid events in the remaining evolved clones increased fitness and (2) generating sufficient data to approach general questions about aneuploidy’s role in adaptation and evolution, we devised a screen to assay the fitness effects of aneuploidy genome-wide.

### Constructing a Genome-Wide Collection of Telomeric Amplicon Strains

In designing our screen, we decided to focus on a particular category of aneuploid event: telomeric amplicons (Tamps), which we defined as a segmental amplification that initiates at a given location in the genome and extends to the proximal telomere. Tamps are a mutation type worthy of focused study as they are frequently observed in our evolved clones (17/36 aneuploid events are Tamps), and Tamps also play a role in human diseases such as cancer and developmental disorders [13,14,38].

**Fig 4:**
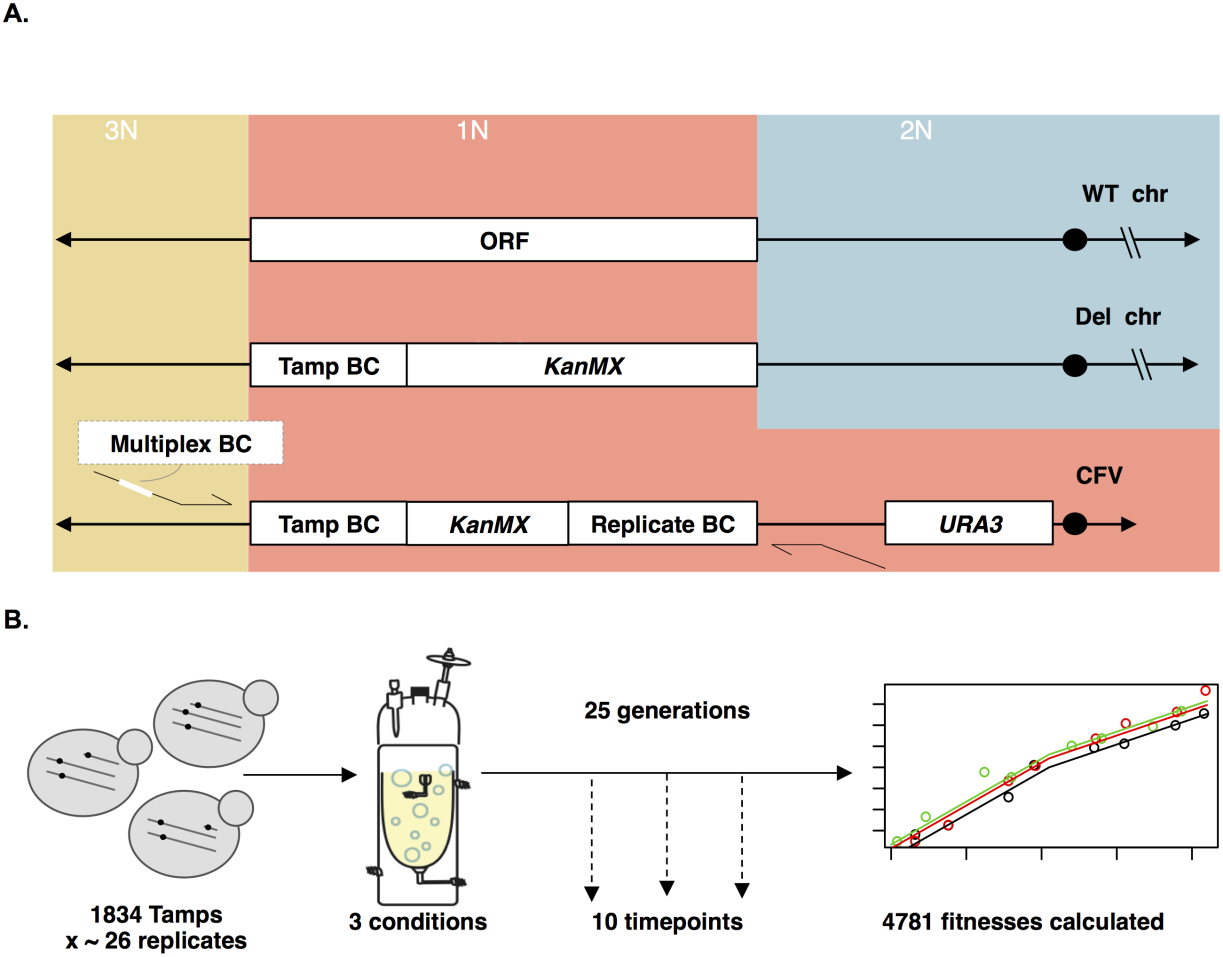
Experimental design for genome-wide screen for the fitness effects of Tamps. A) A genome-wide pool of telomeric amplicon strains (Tamps) was constructed. Each Tamp initiates at the *KanMX* cassette and extends to the proximal telomere, creating a strain that has two chromosomal copies (2N) at most genomic locations, one copy (1N) in the region replaced by the *KanMX* cassette in the deletion collection, and three copies (3N) at locations telomeric of the deleted gene. The Tamp BC and a portion of KanMX are also present at 2 copies. Each strain contains two barcodes: one identifying the Tamp and a second identifying the unique biological replicate. A third barcode was incorporated during the generation of the barcode sequencing libraries which allowed for multiplexing of experimental samples. Large black arrows represent telomeres; large black circles represent centromeres. The primers represent the barseq primers used to create libraries for sequencing. B) Genome-wide pooled competition experimental design.

To construct a genome-wide collection of Tamps, we returned to a classic method of genetic analysis: chromosome fragmentation [39]. This method was originally used for mapping the physical location of cloned genes. In our case, we were interested in it as an approach to fragment the yeast genome into a series of differently sized Tamps. By targeting our chromosome fragmentation vector (CFV) to the *KanMX* cassette that replaces each gene in the yeast deletion collection [40], we were able to generate Tamps initiating at selected genomic locations simply by altering the particular deletion collection strain we chose to transform with our CFV (Fig 4A and S3 Fig). Furthermore, as each deletion collection strain already had a unique DNA barcode identifying the genomic location of the *KanMX* cassette (“Tamp BC” in Fig 4A), we could simply use barcode sequencing (barseq) [28] to determine the location at which the Tamp initiated. The design of our CFV included an additional random 12 base-pair barcode that, upon transformation into the target deletion collection strain, was incorporated into the Tamp and provided a barcode for each independent transformation event of an individual deletion collection strain (Fig 4A “Replicate BC,” see Materials and Methods for details). The ability to track multiple biological replicates of each Tamp allowed us to determine more accurately the fitness for each Tamp.

We chose to build our Tamp pool from the diploid heterozygous deletion collection. We chose this deletion collection for two reasons. First, we wanted to match most closely the diploid background of most of the aneuploid clones isolated from our evolution experiments. Second, we expected there to be fewer suppressor mutations, which are commonly selected for in a homozygous or haploid deletion background to ameliorate the effects of the deleted gene [41,42]. Importantly, we were only able to take advantage of the yeast heterozygous deletion collection in this way because our lab had previously determined a set of 2,254 strains from this collection that have neutral fitness, with a range of relative fitnesses from -0.05 to 0.04, under our standard chemostat growth conditions of sulfate-, glucose-, and phosphate-limitation (S4 Table) [34]. Thus, by restricting our method to these 2,254 deletion collection strains and the limitations under which they have neutral fitness, we can be reasonably confident that any fitness effects we do measure are due to the Tamp itself and not the underlying genetic background.

### A ChrII-Targeted Screen with 21 Tamps Identified Known Driver Genes

With the intent of scaling eventually to the entire genome, we first sought to test our method on a small genomic region carrying a known driver gene: specifically, the telomeric 60 kb on the right arm of chromosome II (chr II). We chose to first focus on this region because it contains the high-affinity sulfate transporter *SUL1*, which our group had previously shown to be advantageous when amplified under sulfate-limiting conditions [10,30]. Furthermore, we demonstrated in the experiments described above that amplification of this region in its native chromosome context is also beneficial (Fig 2, “*SUL1* amp”). We chose 60 kb since that amplicon size is the largest we have observed in diploid sulfate-limited evolution experiments [10]. We hypothesized that only Tamps containing *SUL1* would increase fitness under sulfate-limiting conditions.

**Fig 5:**
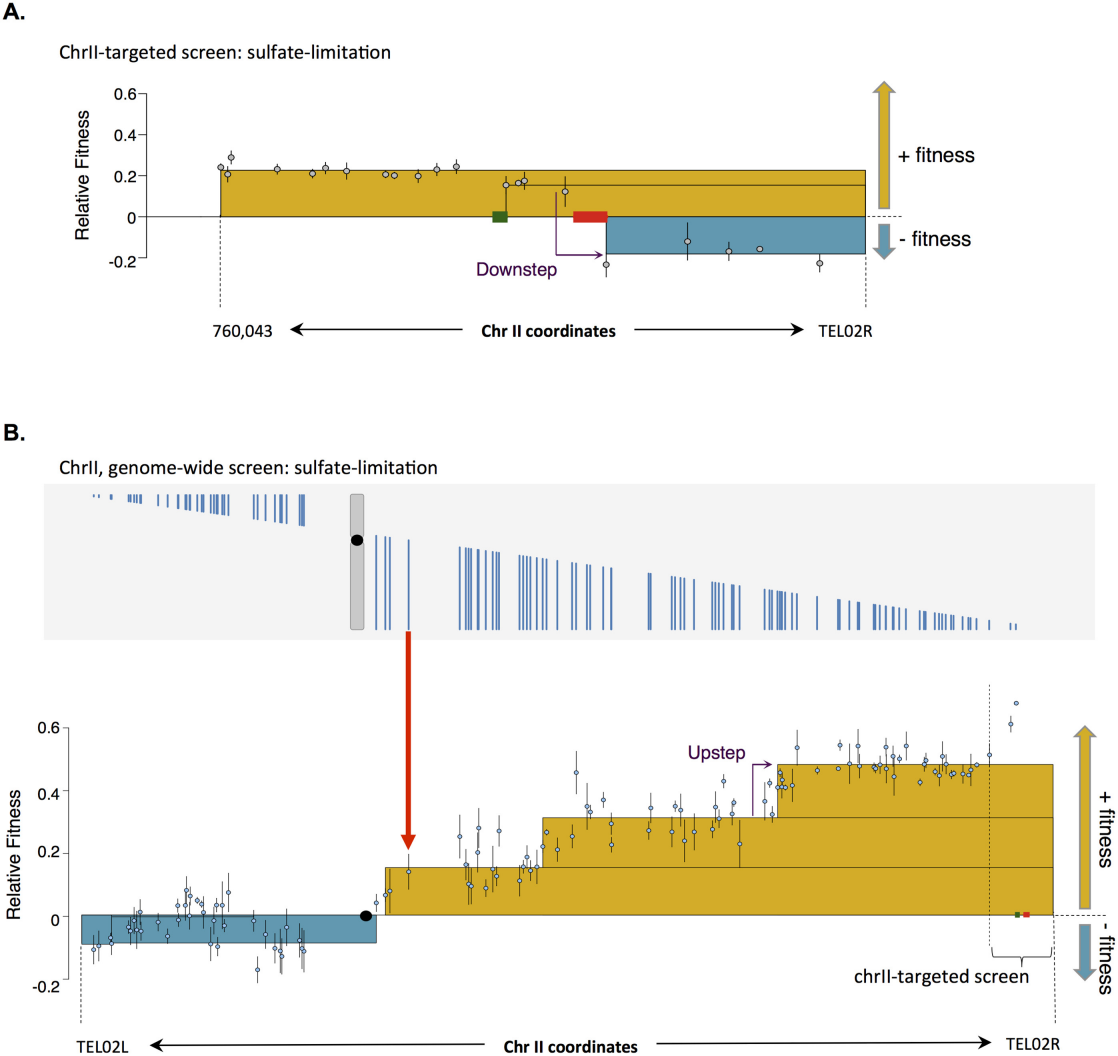
Tamps on the right arm of chromosome II increase fitness under sulfate limitation. A) The chrII-targeted pool of 21 Tamps spanning the right arm of chromosome II identified two driver genes that increased fitness under sulfate-limited conditions: *BSD2* (green rectangle) and *SUL1* (red rectangle). Both *BSD2* and *SUL1* are associated with a Downstep in the fitness landscape. Grey circles represent the individual Tamp fitnesses +/- SE. Each stacked box represents the average fitness of the Tamps enclosed within it; yellow boxes denote positive fitness and teal boxes denote negative fitness. Raw data can be found in S5 Table. B) Top panel: The 122 Tamps spanning chromosome II in the genome-wide screen are represented as vertical blue lines. Bottom panel: the fitness of each Tamp (+/- SE) is plotted as a blue dot directly below the blue line representing the corresponding Tamp; see red guider arrow for an example. The yellow and blue boxes represent the average relative fitness, positive or negative respectively, of the Tamps enclosed. The extent of each box, i.e. each fitness breakpoint, was defined by DNAcopy segmentation of the fitness landscape. Lower panel: As discussed in the text, an example of a Downstep and an Upstep is highlighted with a purple arrow. Please note that as the data presented in parts A) and B) are from two different competition experiments with different pools, the relative fitnesses would not be expected to be identical. Raw data can be found in S6 Table.

We successfully created 21 Tamp strains, each initiating at a different gene within this 60kb region and extending to the right telomere, by transforming 21 neutral-fitness heterozygous deletion strains with our *KanMX-*targeted CFV (see Materials and Methods for additional details). Each deletion strain was transformed individually, and the karyotype confirmed by aCGH (see GEO Accession GSE67769, see Materials and Methods for additional details). Pooled competition of these 21 strains for 9–12 generations followed by sequencing of the deletion collection barcode at five different time points allowed us to track the relative frequencies of each Tamp and infer their relative fitnesses (Fig 5A and S5 Table). As expected, our results demonstrated that Tamps containing *SUL1* increased fitness under sulfate-limiting conditions (Fig 5A). In addition to *SUL1*, a second driver gene had previously been identified within this 60 kb region: *BSD2* amplification increases fitness under sulfate-limiting conditions [34]. Data from this targeted Tamp screen identified both *BSD2* and *SUL1* as driver genes under sulfate-limiting conditions. Tamps containing both *SUL1* and *BSD2* had an average fitness increase of 23%, Tamps containing only *SUL1* had an average fitness increase of 15%, and Tamps containing neither *SUL1* nor *BSD2* had an average fitness decrease of 18%. The same procedure was repeated under glucose-limiting and phosphate-limiting conditions and no increase in fitness relative to wild type was observed (S4 Fig), thus demonstrating the condition-specific fitness effects of the Tamps examined here.

We noticed that both *SUL1* and *BSD2* were highlighted in our data by a discrete decrease in Tamp fitness, or “Downstep” (Fig 5A). This was due to the fact that Tamps lacking *SUL1* or *BSD2* had decreased fitness compared to Tamps containing one or both of those genes. We hypothesized that such Downsteps could be used to identify additional driver genes in our genome-wide screen. Similarly, “Upsteps” could be used to identify genes that increased fitness when no longer amplified on a Tamp. Upsteps, therefore, could be used to identify novel “anti-driver” or growth-inhibiting genes in our genome-wide screen.

### A Genome-Wide Screen Uncovers the Fitness Landscape of Telomeric Amplicons

After we confirmed the validity of our method with the chr II-targeted screen described above, we scaled our approach to the entire genome. The 2,254 neutral-fitness deletion collection strains were pooled and transformed with our *KanMX-*targeted CFV. To ensure each Tamp was represented by multiple independent transformation events, >42,000 transformant colonies were collected, guaranteeing approximately 20 unique biological replicates for each Tamp. Barseq of the resulting Tamp pool revealed it to be of adequate complexity: 1,802 of 2,254 targeted Tamps (80%) were represented by >0.005% of the total reads, and each Tamp was represented by, on average, 26 independent transformants marked by unique biological replicate barcodes.

We next used our genome-wide pool of Tamps to inoculate three glucose-, phosphate-, and sulfate-limited chemostats, for a total of nine pooled competition experiments. Each competition experiment was carried out for approximately 25 generations, with samples for barseq taken at ten time points throughout (Fig 4B). We were able to track the Tamp frequencies of >100,000 unique biological replicates across ten time points under all three conditions. These data allowed us to, after the filtering steps described below, determine the fitnesses of 1,631, 1,596, and 1,551 Tamps in glucose-, phosphate-, and sulfate-limited conditions, respectively (S6 Table).

Our ability to track independent biological replicates of each Tamp was crucial in obtaining accurate Tamp fitness estimates, as our CFV-based method of generating Tamps had a significant error rate: while 20/25 Tamp strains generated in our chr II-targeted pool had the correct karyotype, only eight of the 16 Tamp strains we tested from our genome-wide pool had the correct karyotype as determined by aCGH (see GEO Accession GSE67769). The abnormal Tamp karyotypes included, most commonly, amplicons initiating at the correct genomic location but not extending to the proximal telomere and, occasionally, contained other large aneuploid events (S7 Table). To mitigate the effect of biological replicates for which fitness was mismeasured due to incorrect Tamp formation or background mutations, we first filtered out Tamps with highly variable fitness estimates between biological replicates: this excluded approximately 20% of all Tamps from subsequent analysis and, as summarized above, left 1,631, 1,596, and 1,551 Tamps in glucose-, phosphate-, and sulfate-limited conditions, respectively, for further analysis (see S1 Text for additional details). Next, we combined data from all biological replicates for a given Tamp to obtain a more accurate estimate of each Tamp’s fitness (S5 Fig, See Materials and Methods). Specifically, for those Tamps with more than 15 biological replicates (approximately 55% of remaining Tamps), we used the mode of the fitness distribution described by all biological replicates as the Tamp fitness; when 15 or fewer biological replicates were available, simply the mean of the biological replicates was used as the Tamp fitness (See S1 Text). We confirmed the overall accuracy of our methods in 24 control experiments competing eight Tamp strains in all three nutrient-limiting conditions in head-to-head competition experiments against an appropriate GFP-marked control strain (S8 Table). We found that the fitnesses determined in our genome-wide screen agreed well with those determined in head-to-head competition experiments of aCGH-validated strains (S2B Fig, adjusted R^2^ = 0.64).

When we plotted the fitnesses of each Tamp across the genome, we noticed that, similar to the chrII-targeted screen, neighboring Tamps typically had similar fitnesses, which defined plateaus bordered by distinct fitness breakpoints (S6 Fig and S7 Fig). As described above, we hypothesized that “Downsteps” in fitness could be used to identify driver genes that, under the condition tested, increased fitness when amplified in the context of a Tamp. Similarly, sharp increases in fitness, or “Upsteps,” could be used to identify anti-driver genes that, when amplified in the context of a Tamp, decreased fitness under the tested condition.

After we observed the stepwise shape of this fitness profile, we used DNAcopy [43], an analysis program typically applied to aCGH data to identify regions of similar copy number as well as copy number variant (CNV) breakpoints via circular binary segmentation, to define fitness plateaus and fitness breakpoints in our Tamp fitness data (S9 Table, See Materials and Methods). Segmenting our genome-wide fitness data in this way generated a summary view of the fitness effects of Tamps. We believed this analysis approach was well suited to our data because, similar to CNVs analyzed by aCGH, we expected our fitness data to be somewhat noisy and for neighboring Tamps to have similar fitnesses.

As a good example of our analysis approach, Fig 5B visualizes the results of our genome-wide Tamp screen for chromosome II under sulfate-limiting conditions. The top panel of Fig 5B depicts as blue lines the 122 Tamps spanning chromosome II for which we determined fitnesses; each Tamp initiates at a different location along chromosome II and extends to the proximal telomere. The fitness of each Tamp is plotted in the bottom panel of Fig 5B directly below its corresponding vertical blue line (see the red arrow for one example). Segmenting our genome-wide fitness data using DNAcopy defined fitness breakpoints that are outlined with the yellow and teal stacked boxes: yellow boxes enclose Tamps that increased fitness, while teal boxes enclose Tamps that decreased fitness. Just as we observed in our chromosome II–targeted pool, Tamps that amplified the right arm of chromosome II, where *SUL1* is located, increased fitness under sulfate-limiting conditions. Note that the Downstep telomeric of *SUL1* we observed in the chrII-targeted pool was not observed in the genome-wide pool because we did not include any Tamps initiating between *SUL1* and the telomere in our genome-wide Tamp pool.

Although the incorrect karyotypes of individual Tamp biological replicates is an unfortunate by-product of our methodological approach, our analysis pipeline significantly ameliorated this limitation. We are therefore confident that this genome-wide Tamp screen provided an accurate description of the fitness effects of a complex pool of Tamps. As such, our method provides a systematic view of the fitness landscape described by Tamps under multiple selective conditions.

### Aneuploid Events Affect Fitness in a Condition-Specific Manner

**Fig 6:**
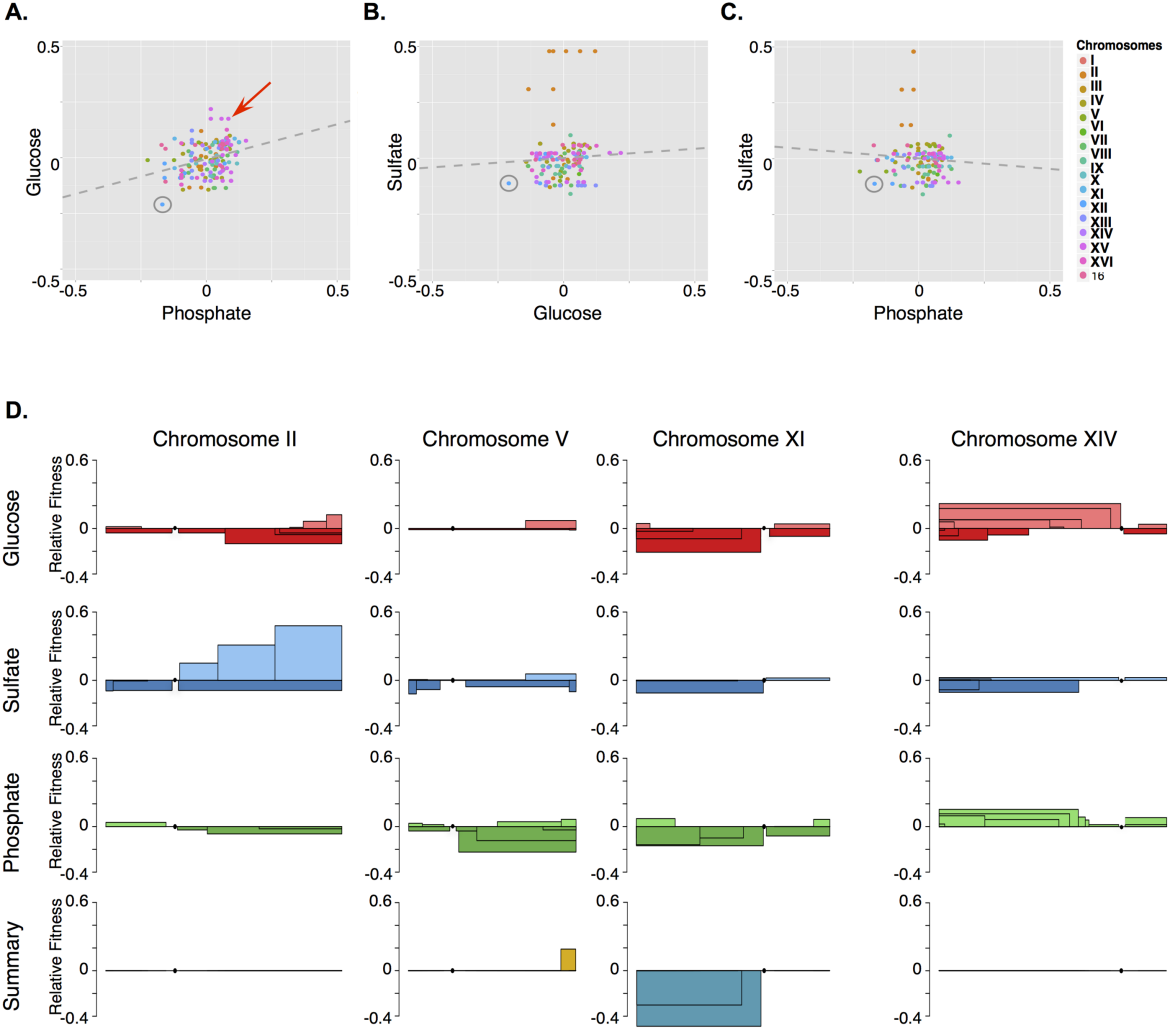
The fitness effects of aneuploidy are typically condition-specific. Each dot in A–C represents one of 175 genomic regions with different fitness in at least one of the three conditions tested (see text for additional details). The adjusted R^**2**^ values are 0.089, -0.003, and -0.002 for A, B, and C respectively. The grey circle indicates a Tamp on the left arm of chromosome XI that decreased fitness under glucose-, phosphate-, and sulfate-limiting conditions. The red arrow indicates a Tamp on the left arm of chromosome XIV that increased fitness under both glucose- and phosphate- but not under sulfate-limiting conditions. D) The stacked boxes represent Tamps with equivalent fitness as determined by the segmentation program DNAcopy (see Materials and Methods for details). The predicted fitness effects from our Tamp screen under glucose-, sulfate-, or phosphate-limiting conditions are shown for four chromosomes including the examples on the right arm of chromosome II and the left arm of chromosome XI (glucose = red, sulfate = blue, phosphate = green). Segments that have a positive or negative fitness in all conditions are indicated in the summary panel at the bottom of part D as yellow or teal boxes respectively. Raw data can be found in S9 Table.

Next, we asked whether the fitness effects of Tamps were always condition dependent or if there were some Tamps that commonly increased or decreased fitness across the conditions we examined. Our genome-wide Tamp screen identified a unique list of fitness breakpoints in each of the three conditions we examined. The union of these three lists thus defines the minimum number of regions showing a change in fitness compared to neighboring regions in at least one condition. Specifically, we identified 175 regions with different fitnesses in at least one condition. We compared the fitnesses of these 175 regions between conditions and generally found little correlation between conditions (Fig 6A–6C). However, a few regions had common fitness effects between conditions; four and seven of the 175 regions increased or decreased fitness by >5%, respectively, in all three conditions.

As examples of our Tamp dataset, the fitnesses of Tamps from four chromosomes are shown in Fig 6D. Similar to Fig 5B, in this figure we have shown stacked boxes that represent groups of Tamps with similar fitness as defined by our segmentation of the genome-wide fitness profile with DNAcopy. However, in this figure we have not plotted raw Tamp data as we did in Fig 5. As we were interested in comparing the fitness effects of Tamps between conditions, we summed the fitness effects of each Tamp under all three conditions. To emphasize regions that have common effects between all three conditions, in the “Summary” section of Fig 6D we displayed as stacked boxes only those Tamps with the same fitness effect under all three conditions (i.e., >5% fitness advantage or disadvantage). The relative fitness of these boxes represents the sum of the fitness effects under all three conditions. Notice that some of the stacked boxes appear to be missing from the”Summary” section of Fig 6D. This is because only a few regions had common fitness effects between all conditions; boxes enclosing regions with different fitness effects under different conditions are excluded from the “Summary” section.

While chromosome II and XIV lacked any region with a common fitness effect across all three conditions, chromosomes V and XI both contained regions that were either universally advantageous or detrimental when amplified. For example, amplification of the left arm of chromosome XI decreased fitness under all three conditions (Fig 6A–6C, grey circles, and Fig 6D). Other Tamps showed common fitness effects in two of the three conditions we tested. For example, amplification of the left arm of chromosome XIV increased fitness not only under glucose-limiting conditions but also under phosphate-limiting conditions (Fig 6B, red arrow, Fig 6D).

Next, we examined two regions recurrently amplified in the set of evolution experiments examined here and those previously described [12]. The right arm of chromosome V was amplified in three different evolution experiments carried out under the three nutrient-limiting conditions. Similarly, the genome-wide Tamp screen predicted a 51 kb Tamp on the right arm of chromosome V to increase fitness by approximately 6%–7% under all three conditions (Fig 6D). However, the Tamps observed in the evolved populations were actually somewhat larger (84–440 kb) than this 51 kb Tamp. It is notable that the chromosome V amplicon in two of the three evolved populations initiated at the closet Ty element centromeric of this 51 kb high-fitness Tamp. The Tamp screen predicted the chromosome V amplicons observed in the evolved populations to affect fitness by +6%, -3%, and -1% under sulfate-, phosphate-, and glucose-limiting conditions respectively (S10 Table). In summary, while our Tamp screen predicted that amplification of 51 kb on the right arm of chromosome V is commonly advantageous, the precise amplifications observed in our evolution experiments were predicted to be neutral under glucose- and phosphate-limiting conditions and to increase fitness only under sulfate-limiting conditions.

The recurrent amplification on the right arm of chromosome XIV has been observed in three independent glucose-limited evolution experiments [10,12]. Consistent with these observations, the genome-wide Tamp screen predicted this event to increase fitness by >20% under glucose-limited conditions (Fig 6D). Interestingly, our genome-wide screen predicted a smaller Tamp on the left arm of chromosome XIV to increase fitness under phosphate-limiting conditions; however, no such amplicon has been yet reported in any phosphate-limited evolution experiment. Chromosome XIV left-arm Tamps were predicted to have a nearly neutral effect on fitness under sulfate-limiting conditions (< 2% fitness increase). A similar rearrangement was also previously identified as yeast “chromosome XVII” because of an aberrant karyotype in the original genetic mapping strains, suggesting this amplification may have fitness benefits in other conditions as well [44].

### The Genetic Basis for Aneuploidy’s Effect on Cellular Fitness

The dataset from our Tamp screen allowed us to ask general questions about the relationship between aneuploidy, specifically telomeric amplicons, and fitness. First, we compared the fitness of each Tamp to its size in base-pairs and found little correlation (adjusted R^2^ = 0.05, S2C Fig). Although Tamp truncation was not an insignificant problem in our dataset, our analysis approach, by filtering out Tamps with high intra-replicate variation in fitness and using the mode of the biological replicate fitness distribution to estimate fitness, partially ameliorated the effects of incorrectly sized replicates on each Tamp’s fitness estimate.

**Fig 7:**
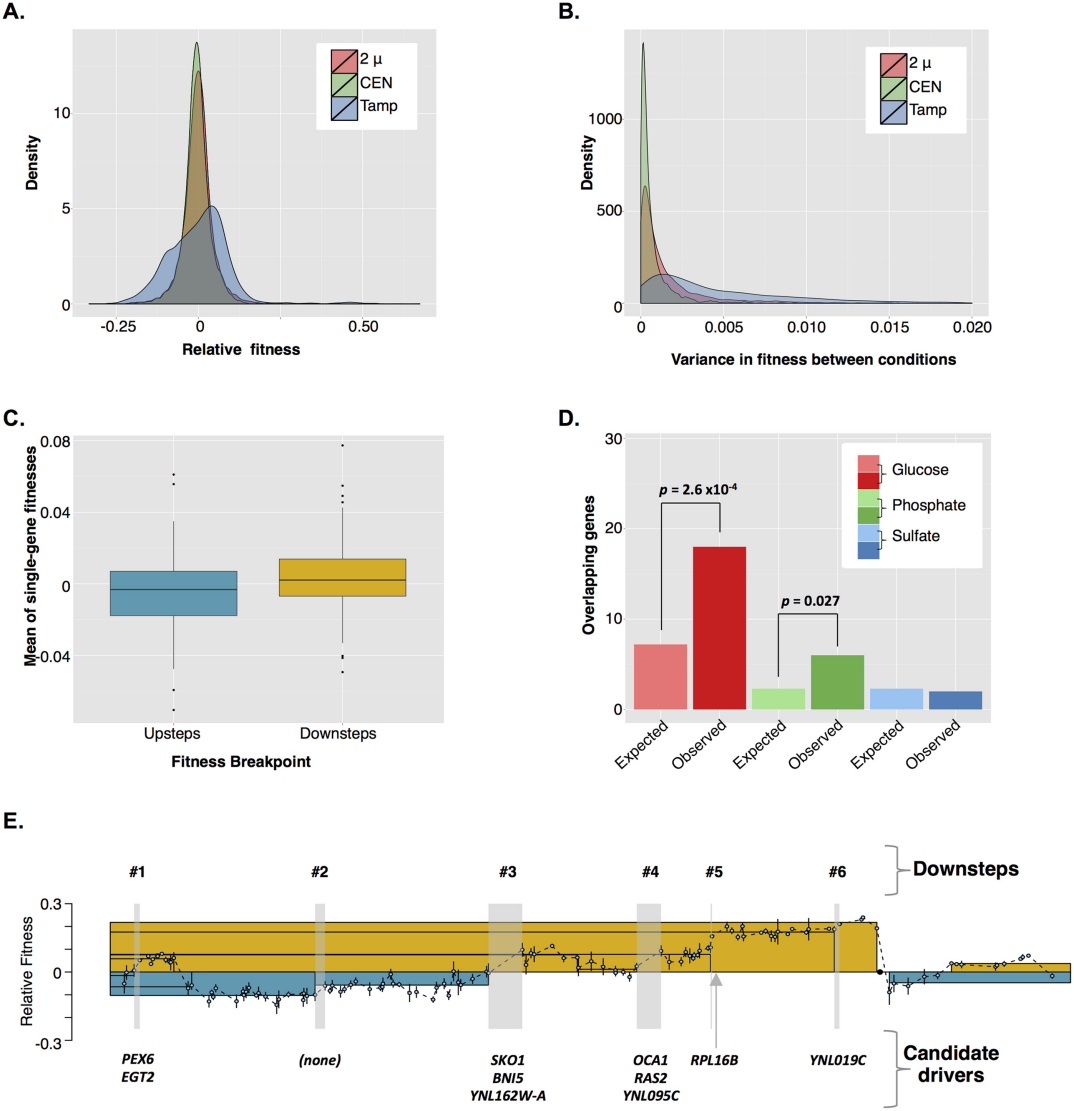
The genetic basis for aneuploidy’s effect on cellular fitness. A) The fitness landscape explored by Tamps is much broader than that explored by single-gene amplification. CEN = fitness density distribution of a genome-wide collection of yeast strains with each gene cloned into a low-copy-number CEN plasmid (raw data from [34], S2 Table), 2 μ = fitness density distribution of a genome-wide of yeast strains with each gene cloned into a high-copy-number 2 μ plasmid (raw data from [34], S2 Table), Tamp = fitness density distribution of Tamp as determined by the Tamp screen described in this study (raw data from this study S6 Table). B) Tamps are more pleiotropic than single-gene amplifications. Pleiotropy is defined here as the between-condition variance in fitness [See S1 Text]. CEN = density distribution of variance in fitness of a genome-wide collection of yeast strains with each gene cloned into a low-copy-number CEN plasmid (raw data from [34], S2 Table), 2 μ = density distribution of variance in fitness of a genome-wide collection of yeast strains with each gene cloned into a high-copy-number 2 μ plasmid (raw data from [34], S2 Table), Tamp = density distribution of variance in fitness of Tamps as determined by the Tamp screen described in this study (raw data from this study S6 Table). C) The average fitness effects of all single-gene amplifications overlapping a fitness breakpoint is greater for Downsteps than for Upsteps (unpaired, two-tailed *t* test, *p* = 0.008). Raw data for individual single-gene amplifications is from [34], S2 Table. Raw data can be found in S17 Table. D) Upsteps in glucose- and phosphate-limiting conditions are enriched for genes mutated in glucose- and phosphate-limited evolution experiments. Raw data can be found in [34], S4 Table; from this study, Upstep and Downstep genes can be found in S17 Table. E) Our Tamp screen predicted amplification of the left arm of chromosome XIV to increase fitness under glucose-limiting conditions. Our Tamp screen identified six Downsteps along this region, all but one of which have a candidate driver gene associated with them. The yellow and blue boxes represent the average relative fitness, positive or negative, respectively, due to amplification of the region enclosed. Raw data can be found in S6 Table, S9 Table, and S11 Table.

Next, we took advantage of data previously generated by our lab that determined the fitness effects of single-gene amplifications genome-wide under the same conditions explored in our Tamp screen [34] (see S1 Text). We compared the fitness distribution defined by our genome-wide Tamp screen to the fitness distribution defined by single-gene amplifications [34] (Fig 7A). We found that the distribution of Tamp fitnesses was much broader than that defined by single-gene amplifications. Additionally, we noted that distribution of Tamp fitnesses appeared bimodal, with one negative fitness peak and a second positive fitness peak. This result supports the hypothesis that aneuploid events are mutations that have large effects, positive and negative, on organisms’ fitness.

Aneuploid events are hypothesized to be highly pleiotropic: a characteristic that may explain their eventual supplantation by more targeted mutation types [37]. To test this hypothesis, we defined pleiotropy as the between-condition variance in fitness. Taking advantage of the same genome-wide single-gene amplification dataset referenced above [34], we compared the density distributions of variance in fitness of Tamps to those of single gene amplifications. We found that Tamps described a much broader distribution than that described by single gene amplification (Fig 7B). These results support the hypothesis that aneuploid events are pleiotropic.

The data from our genome-wide Tamp screen, combined with our lab’s previous data describing the fitness effects of single-gene amplifications genome-wide, also allowed us to explore the genetic basis of aneuploidy’s effects on cellular fitness. First, we asked if the fitness of any given Tamp could be predicted by the average of the fitness effects of all single-gene amplifications within the boundaries of the Tamp. We found that the average of the fitnesses of single-gene amplifications for the genes contained within a Tamp did not predict the fitness of the Tamp itself (S2D Fig) Next, we explored the alternative hypothesis that only a few genes within a Tamp are centrally important for effecting the fitness of the entire amplicon. This hypothesis was additionally supported by the stair-step shape of the Tamp fitness landscape: if many genes within a Tamp contributed to the fitness effects observed, one would expect a smooth fitness profile in which the addition or loss of individual genes from the amplicon produced an incremental change in fitness; instead, the fitness profile produced by our Tamp screen often revealed plateaus in fitness bordered by discrete fitness breakpoints. As discussed above, we hypothesized that Downsteps in the Tamp fitness landscape identified driver genes that increased fitness when amplified, while Upsteps identified anti-driver genes that decreased fitness when amplified. Combining fitness data from all three conditions, we identified 181 fitness breakpoints: 77 Downsteps and 104 Upsteps. As our genome-wide Tamp screen did not contain a Tamp for every gene in the genome, each Downstep or Upstep region necessarily overlapped several genes. We averaged the fitness effects of single-gene amplification for genes overlapping each of the 181 fitness breakpoints and found that the average fitness at Downsteps was significantly greater than at Upsteps (Fig 7C, unpaired, two-tailed *t* test, *p* = 0.008, raw data in S17 Table). These results supported the hypothesis that one or few gene(s), located at Upsteps and Downsteps, were primarily responsible for effecting the fitness of each Tamp.

By identifying driver and anti-driver genes respectively, Downsteps and Upsteps can be used to identify potential genetic targets of adaptation. In fact, under both glucose- and phosphate-limiting conditions, but not under sulfate-limiting conditions, the genes overlapping Upsteps were enriched for genes mutated in populations evolved under the corresponding nutrient limitation (Fig 7D, Fisher’s exact test, *p* = 2.6 x 10^–4^ and *p* = 0.027 for glucose- and phosphate-limiting conditions, respectively) [34]. As we expected Upstep genes to decrease fitness when amplified, we might therefore have expected that lower levels of expression of these same genes would increase fitness. Our results thus agree with the recent observation by Kvitek and Sherlock that the majority of mutations selected in haploid yeast evolved under glucose-limited conditions are loss-of-function mutations [45]. There are additional similarities between the glucose-limited Upstep genes identified in our Tamp screen and the genes mutated in glucose-limited evolution experiments. First, glucose-limited Upstep genes are enriched for Gene Ontology (GO) terms closely related to those enriched in the group of recurrently mutated genes identified by Kvitek and Sherlock in glucose-limited evolution experiments (“intracellular signal transduction,” Fisher’s exact test, Holm–Bonferroni corrected *p* = 0.01, and “regulation of intracellular signal transduction,” Fisher’s exact test, Holm–Bonferroni corrected *p* = 0.015). Second, the genes located at Upsteps in our glucose-limited Tamp screen are enriched for genes observed by Kvitek and Sherlock to be recurrently mutated in glucose-limited evolution experiments and include: *HOG1*, *IRA2*, *LCB3*, *PBS2*, *PDE2*, and *SSK2* (Fisher’s exact test, *p* = 6.4 x 10^–6^).

Given the large number of genes overlapping several Downsteps (up to 30 genes), we sought to filter the list of Downstep genes and produce a list of high-quality candidate driver genes. We filtered the list of Downstep genes by comparing it to several published datasets: the list of genes commonly up-regulated in clones evolved under glucose-, phosphate-, or sulfate-limiting conditions [10,46]; the list of genes that increased fitness when present on a low-copy-number plasmid under glucose-, phosphate-, or sulfate-limiting conditions [34]; and the list of genes mutated in populations evolved under glucose-, phosphate-, or sulfate-limiting conditions [34]. After this filtering, we identified a total of 100 candidate driver genes important for increasing fitness in the context of a telomeric amplicon under the three nutrient-limiting conditions we examined here (S11 Table). Importantly, our filtered list of candidate driver genes still identified at least one driver gene at most Downsteps (58 out of 77, or 75%).

Although we expected our method to identify driver genes that, when amplified, individually increased fitness, we also expected our method to identify genes that increased fitness only when amplified in the context the Tamp. In fact, 12 of the 73 candidate driver genes identified here have a negative effect on fitness when amplified individually (S11 Table). We hypothesized that these 12 driver genes in particular must synergistically interact with one or more genes coamplified on the Tamp. The synergistic partners of the identified driver genes are probably located between the identified driver gene and the telomere. As these telomeric synergistic partners would only be expected to affect fitness when coordinately amplified with our currently identified driver genes, they would not be expected to produce a step in the fitness profile. Identification of these pairs or groups of synergistically interacting genes remains a target of future research.

With the fitness data from the genome-wide Tamp screen and this list of candidate driver genes in hand, we returned our analysis to the aneuploid events observed in our laboratory-evolved populations as well as aneuploid events previously documented in a similar set of evolution experiments [12]. Fitness data from our Tamp screen predicted that 11 of the 16 telomeric amplicons identified in evolved populations increased fitness under their corresponding conditions, while the remaining five telomeric amplicons were likely passenger mutations (two of these five amplicons represented the chromosome V amplicons observed in sulfate- and phosphate-limited populations and discussed above) (S10 Table). Importantly, our Tamp screen allowed us to predict the fitnesses of telomeric amplicons that are difficult to test by traditional genetic means, as they are linked to large deletions that rendered any haploid spore intermediary inviable. For each telomeric amplicon observed in an evolved population, we estimated the number of driver genes within its length by counting the number of Downsteps it overlapped (S10 Table). Typically, evolved Tamps overlapped one to three Downsteps, suggesting that only a few driver genes were primarily important for determining the fitness increase associated with these aneuploid events. As mentioned above, we have not yet identified the synergistic partners of these driver genes. The main exception to this statement is the amplification of the left arm of chromosome XIV recurrently observed in populations evolved under glucose-limiting conditions (Fig 7E). This large amplification overlapped six Downsteps and was predicted by our screen to increase fitness by >20%. There were multiple candidate driver genes along this segment, including *YNL019C*, *RPL16B*, *OCA1*, *RAS2*, *YNL095C*, *SKO1*, *BNI5*, *YNL162W-A*, *PEX6*, and *EGT2*.

The data from our Tamp screen proved useful in addressing general questions about the genetic basis for aneuploidy’s effect on cellular fitness and identified potentially novel driver genes that are important for increasing fitness in the context of aneuploidy. Furthermore, we have used data from our Tamp screen to predict the fitness effects of telomeric amplicons observed in evolved populations that are otherwise not amenable to traditional genetic analyses.

## Discussion

### Aneuploidy Affects the Adaptation and Evolution of Asexually Dividing Cells

Our survey of aneuploid events identified in populations of *S*. *cerevisiae* evolved in nutrient-limited chemostats produced circumstantial evidence for aneuploidy’s positive effect on cellular fitness: aneuploid events rose to high population frequencies, and clones isolated with aneuploid karyotypes had fitnesses greater than wild type. In addition, we found that evolved aneuploid clones had a significantly greater relative fitness than evolved euploid clones. However, as the aneuploid and euploid clones were also different with respect to their genetic background, the nutrient-limiting conditions of their evolution experiment, and the number of generations that they were grown in the chemostat [10], there are several possible confounding explanations for their significant difference in fitness.

Three out of four aneuploid events, for which we directly determined the fitness, were sufficient to increase fitness relative to wild type. Each, however, showed a different relationship to the overall fitness of the original corresponding evolved clone, demonstrating that aneuploid events show varying degrees of epistasis with the other mutations acquired over the course of evolution. Interestingly, the V_R_ t X_CEN_ supernumerary chromosome isolated from the sulfate-limited population S8, despite occupying a substantial proportion of the population (13%), decreased fitness under sulfate-limiting conditions. Furthermore, this supernumerary chromosome contained a telomeric amplification of the right arm of chromosome V that was recurrently amplified in three different populations evolved under three different nutrient-limited conditions. Both the population frequency of this event as well as its recurrence were strongly suggestive of its selection under sulfate-limiting conditions. However, the S8c2 V_R_ t X_CEN_ supernumerary chromosome actually decreased fitness by 10% under sulfate-limiting conditions. It is possible that this discrepancy can be explained by the non-transitive relationship of fitness that has previously been observed over the course of laboratory evolution [47]. Epistasis may also explain this result, as the *SUL1* amplicon alone from clone S8c2 increased fitness to a similar extent as that observed with the original evolved clone; this suggests that the fitness effects of the V_R_ t X_CEN_ supernumerary chromosome were fairly neutral in the context of a *SUL1* amplification. These results argue that the V_R_ t X_CEN_ supernumerary chromosome is a passenger mutation. This is consistent with previous findings that showed genetic hitchhiking to be important to the spectrum of mutations observed in populations of asexually dividing cells [33,48]. Given the strong effects of epistasis and genetic hitchhiking on mutation frequency, these results should offer a strong cautionary message to the sole reliance on recurrence and population frequency for differentiating driver mutations from passenger mutations.

Although the remaining telomeric amplicons observed in the laboratory-evolved populations examined in this study were all concurrent with large deletions, making their genetic isolation difficult using traditional techniques, data from our Tamp screen allowed us to predict that the majority of these laboratory-evolved Tamps increased fitness in the conditions under which they were observed. In contrast, only one out of the 12 non-synonymous mutations we tested, a missense mutation in *PHO84* isolated from the phosphate-limited evolved clone P6c1, increased fitness by more than 5%. Consistent with this observation, we observed a broader range of fitness effects in our Tamp screen than in a genome-wide screen for the fitness effects of single-gene amplifications (Fig 7A) [34]. These results show that aneuploid events are important drivers of increased fitness in populations of *S*. *cerevisiae* evolving under nutrient limiting conditions. Furthermore, these data are consistent with the hypothesis that aneuploid events allow evolving populations to broadly explore a fitness landscape by prompting large jumps in fitness unattainable by the mutation of single genes [23,37]. Aneuploidy is likely particularly important for the adaptation to novel conditions.

### The Fitness Effects of Aneuploidy Are Typically Condition-Specific

Fitness data from our Tamp screen and from competition experiments with aneuploid events and evolved aneuploid clones confirmed that Tamps, and aneuploidy more generally, are pleiotropic mutations with typically condition-dependent fitness effects; most aneuploid events and clones had decreased fitness under alternative conditions. Occasionally, as observed in both the Tamp screen and in direct fitness assessments with evolved clones and evolved aneuploid events, similar fitness effects were observed between conditions. Notably, similar fitness effects under different conditions were observed with the V_R_ t VI_R_ supernumerary chromosome isolated from the phosphate-limited population P6, which increased fitness to a similar extent under both phosphate- and glucose-limiting conditions. Particularly surprising was the observation that the supernumerary chromosome isolated from the sulfate-limited population S8, which decreased fitness by 10% under sulfate-limiting conditions, actually increased fitness by 11% under glucose-limiting conditions. As all of the competition experiments were carried out under conditions of chemostat growth, it is possible that some of the aneuploid events with common fitness effects across nutrient-limiting conditions affected growth under continuous culture generally.

Mutations such as the S8 supernumerary chromosome might contribute to the increased adaptability of aneuploid cells: an aneuploid event acting as a passenger mutation under a cell’s current condition could provide a dramatic increase in fitness under a novel condition. This conversion of a passenger mutation to a driver mutation may be more likely to occur with aneuploid events than with point mutations or single-gene changes in copy number because of the number of genes affected by a single aneuploid event. For example, although not yet observed in phosphate-limited evolution experiments, our Tamp screen predicted the amplification of the left arm of chromosome XIV to increase fitness under both glucose- and phosphate-limiting conditions (Fig 6D). However, only one of the ten candidate driver genes present within this amplicon is predicted to be responsible for the increased fitness of this amplicon under both phosphate- and glucose-limiting conditions. By affecting the copy number of many genes simultaneously, aneuploid events are necessarily pleiotropic. However, while aneuploid events may also show similar fitness effects under different conditions, these fitness effects are likely mediated through the copy-number change of distinct groups of driver genes. These data emphasize the condition-dependent nature of aneuploidy’s effect on cellular fitness and may help address the “aneuploidy paradox”: the observation that while aneuploidy typically decreases a cell’s proliferative ability, it increases fitness under certain conditions [2,3,5,7].

### The Fitness Effects of Telomeric Amplicons

The data from our Tamp screen allowed us to investigate general aspects of the relationship between aneuploidy and cellular fitness. The data presented here are further support for the current hypotheses that aneuploidy is both a large effect-size mutation and that it is more pleiotropic than single-gene changes in copy number. As aneuploidy generally decreases cells’ proliferative ability, one might have expected larger Tamps to increase fitness to a lesser extent than smaller Tamps as the burden of carrying such a large Tamp outweighed any benefit due to amplification of genes along its length. However, our Tamp data show that there is no overall correlation between size and fitness of the Tamps examined in our screen. Although there was no overall correlation between fitness and Tamp size in our data, there was a distinct negative relationship between size and Tamp fitness on the right arm of chromosome II under sulfate-limiting conditions (slope = -0.0087 relative fitness/kb, adjusted R^2^ = 0.87). A more detailed analysis of the fitness data produced by our Tamp screen may reveal a more general relationship between Tamp size and fitness. In addition, as it is likely that Tamps initiating far from a telomere are less likely to complete break-induced-replication (BIR), thus resulting in truncated amplicons, our results here, while ameliorated by our analysis pipeline, likely represent an underestimate of any deficit correlated with size.

Our Tamp screen revealed that amplicons with increased fitness could not be differentiated from amplicons with decreased fitness simply by averaging the fitness effects of all single-gene amplifications along their lengths. However, when we focused on fitness breakpoints, we were able to differentiate increases in fitness (Upsteps) from decreases in fitness (Downsteps) by averaging the fitness effects of all single-gene amplifications overlapping the breakpoint region. These results suggested that a minority of genes were responsible for an amplicon’s fitness effects. In fact, we have previously shown this to be true for Tamps of the right arm of chromosome II and the amplification of *SUL1* under sulfate-limiting conditions (increase in fitness due to *SUL1* on a low-copy number plasmid = 23% [30], increase in fitness due to 60 kb telomeric amplicon overlapping *SUL1* = 16%). However, the amplification of *SUL1* under sulfate-limiting conditions is a clear outlier, in that it increases fitnesses much more than any other single-gene amplification under the three nutrient-limited conditions examined here. Synergistic effects between the driver genes identified in this study and a small number of as-yet-unknown interaction partners located distally along the amplicon are likely responsible for the fitness effects of most amplicons.

While the models tested here limited the interaction between genes within an amplicon to be simply additive, we acknowledge that the interactions between genes within aneuploid regions are likely to be much more complex and warrant further study. In fact, we have already tested a method to confirm the identity of driver genes and reveal synergistic partners within a Tamp. Focusing once more on the right arm of chromosome II, we created 21 independent strains that each paired a single 60 kb Tamp with deletion of a different gene along this amplicon. As expected, under sulfate-limiting conditions, deletion of *SUL1* eliminated the fitness increase due to this 60 kb Tamp (S8 Fig). Genome-wide application of this method, or focused application to amplicons of particular interest, for example the amplification of the left arm of chromosome XIV under glucose-limited conditions, would further illuminate the types of interactions between genes contained within aneuploid regions.

### Constructing a Genome-Wide Collection of Tamps: Advantages and Limitations

While the data produced by our Tamp screen have allowed us to gain a genome-wide view of the fitness landscape explored by Tamps, it is prudent to highlight some of the limitations of this dataset. First, as noted above, there is a high error rate in the formation of Tamps with the method employed here. Despite our attempts to account for these errors in both our experimental design (i.e., incorporating a biological-replicate barcode into each Tamp strain) and analysis pipeline, future experiments would benefit from an improved experimental approach. One approach would be to identify replicate barcodes that were associated with Tamps of inappropriate sizes and exclude these barcodes from the analysis. This could be accomplished by pairing Pulse-Field Gel Electrophoresis with gel extraction and barseq to determine the actual size of each barcoded Tamp strain. Second, the segmentation approach used to fragment the genome-wide Tamp fitness data into regions of approximately equal fitness may be an oversimplification of these data, and a more detailed examination of the fitness changes across the genome is warranted.

### Amplicons from Evolved Populations Are Constrained by Genomic Context

Our genome-wide Tamp pool represents all possible amplicons at 3–4 gene resolution, however, previous studies have shown that rearrangements, including those that produce the Tamps studied here, are often mediated by repetitive elements in the *S*. *cerevisiae* genome, such as Ty elements [12]. As such, it becomes informative to compare the telomeric amplicons observed in evolution experiments to our Tamp screen and ask if the most advantageous Tamps are selected during the course of laboratory evolution. The recurrent amplicon of the right arm of chromosome V provides a good example of the benefits of this analysis. As described above, the chromosome V amplicon observed in both phosphate- and sulfate-limited evolution experiments is larger than the highest-fitness Tamp on the right arm of chromosome V identified in our screen. This is likely due to the fact that there are no Ty elements or repetitive regions closer to the fitness breakpoint identified by our Tamp screen than the one employed to form the amplicons observed in the evolution experiments. This provides an example where the genomic context likely restricted the formation of the most advantageous amplicon under these conditions.

Generally, however, the regions commonly amplified in evolution experiments are the highest-fitness Tamps identified by our screen. The top 54 most advantageous Tamps identified by our screen under sulfate-limiting conditions all overlap the *SUL1-*containing region on the right arm of chromosome II. Similarly, amplification of the left arm of chromosome XIV has been observed repeatedly under glucose-limiting conditions; 15 of the top 16 most advantageous Tamps identified by our screen under glucose-limiting conditions overlap the left arm of chromosome XIV. Unlike sulfate- and glucose-limited evolution experiments, populations evolved under phosphate-limited conditions show no such obvious recurrent amplification. However, the most fit Tamp predicted by our screen is the amplification of the left arm of chromosome XVI, although of a slightly smaller size than that predicted to be advantageous under glucose-limiting conditions (Fig 6). Although this amplification has not yet been observed in any phosphate-limited evolution experiment to date, there are several Ty elements in the region where positive-fitness Tamps initiated in our screen, so its absence cannot easily be explained by a genomic context unfavorable to amplification.

By combining data from a genome-wide telomeric amplicon screen and detailed analyses of clones and aneuploid events isolated from laboratory evolution experiments, we have provided details about the relationship between aneuploidy and cellular fitness. These data identified new candidate driver genes, the copy number changes of which are important for fitness, contribute to our understanding of how aneuploidy acts at the cellular level, and add to our understanding of aneuploidy’s role in adaptation and evolution. Recent advances in the direct targeting of DNA breaks in human cells [49], combined with the wealth of information generated from the sequencing of cancer genomes, may allow a similar comparative approach to be applied to the effects of aneuploidy in cellular proliferation and its role in the evolutions of cancers. We also note that the same experimental design could be applied to conditions in which aneuploidy is known to be detrimental as a way to identify critical dosage-sensitive genes.

## Materials and Methods

### Strains, Media, and Primers

The strains, plasmids, and primers used in this study are listed in S12 Table, S13 Table, and S14 Table, respectively. Unless specified below, yeast strains were grown at 30°C and standard media recipes were used.

### aCGH to Determine Population Frequency of Aneuploid Events in Evolved Populations

We generated population DNA from archived glycerol stocks for the 14 evolution experiments not previously examined and determined the population frequency of aneuploid events by aCGH. We confirmed the accuracy of this approach by comparing the population frequencies of aneuploid events in population P7, as determined previously in [10] from fresh population DNA samples, to the frequencies determined by the method described here and found similar results. All aCGH data are available at GEO Accession GSE67769. In addition, we used a PCR assay that amplified the breakpoint of the V_R_ t X_CEN_ translocation event present in population S8 using primers OAS005–0AS0008. This breakpoint PCR assay identified the V_R_ t X_CEN_ supernumerary chromosome in 13 of 98 total clones tested (13%); our population aCGH determined the frequency of V_R_ t X_CEN_ supernumerary chromosome to be 13%.

### Chemostat Competition Experiments to Determine Relative Fitness

To determine relative fitness, we competed individual clones of test strains against an appropriate control strain with eGFP integrated at the HO locus in nutrient limited chemostats. We used both large volume (approximately 300 ml) and small volume (20 ml) [50] chemostats for competition experiments. A single colony of each control or test strain was used to start an overnight culture in the same media in which the competition experiment was to be carried out; the overnight culture was then grown at 30°C for approximately 12–36 h. 1 ml of this overnight was used to inoculate each chemostat, which was then allowed to grow at 30°C without dilution for approximately 30 h, at which point fresh media was added to the culture chamber at a rate of 0.17 hour^-1^. After achieving steady-state, 50% of a control-strain chemostat was mixed with 50% of a test-strain chemostat, resulting in two chemostat replicates for a single competition experiment. Flow-cytometry using a BD Accuri C6 flow cytometer (BD Biosciences) at regular intervals throughout the competition allowed us to track the percentage of GFP-marked control cells over time. The data were plotted with ln[(dark cells/GFP+ cells)] versus generations, and we defined the slope of this relationship as the relative fitness of the test strain. The number of replicate competition experiments, as well as the appropriate control strain, is detailed for all test strains in S2 Table.

### Construction of Targeted Tamps by Individual Transformation

Two Tamp strains were constructed individually by direct transformation with a chromosome-fragmentation vector (CFV). 250 bp of homology to the genomic location at which we desired to create a Tamp was cloned into the multiple cloning site of the previously designed CFV YCF4 [39]. The appropriate CFV was then transformed into a haploid FY background strain to create chrIIR-Tamp 1N and chrVR-Tamp 1N and the karyotype confirmed by aCGH (see GEO Accession GSE67769). These haploid strains were backcrossed to create chrII-Tamp 2N and chrVR-Tamp 2N.

### Illumina Sequencing of Evolved Clones and Populations

DNA samples from evolved clones and populations were prepared for WGS using Illumina Nextera kits according to the provided protocol. Libraries were sequenced on either an Illumina HiSeq or a GAII, generating the number of reads detailed in S3 Table. Reads were aligned with BWA [51] and SNVs were called using samtools [52] after applying standard filters. Population frequency of SNVs from population samples was determined from the allele frequency displayed in Integrative Genome Viewer (IGV) [53]. The clones and populations analyzed here (P6c1, P6, P5c3, P5, S8c2, and S8) were included in a previous analysis [34] and the raw data are deposited at BioProject ID PRJNA248591 and BioSample numbers SAMN02800460 (S8c2), SAMN02800438 (P6c1), SAMN02800436 (P5c3, run 1), SAMN02800435 (P5c3, run 2), SAMN02800403 (CEN.PK WT diploid, run 1), and SAMN02800404 (CEN.PK WT diploid, run 2).

### Backcrossing to Isolate Aneuploid Events and SNVs from Evolved Clones into Wild-Type Background

To isolate individual mutations (both SNVs and aneuploid events) identified by WGS of the evolved clones P6c1 and P5c3, we backcrossed each evolved clone to an isogenic wild-type strain of the opposite mating type, sporulated, and dissected tetrads using standard sporulation media and protocols. Evolved clone S8c2, a diploid, was itself sporulated and tetrads dissected using standard sporulation media and protocols. After Sanger-sequencing confirmed the SNVs identified by WGS, tetrads were genotyped by Sanger sequencing and backcrossed repeatedly until each SNV and aneuploid event was isolated into an otherwise wild-type background. Spores isolated from S8c2 with the desired genotype were backcrossed a final time so that each mutation was once again in a diploid background. The karyotypes were confirmed by aCGH for all clones eventually used for relative fitness competition experiments (see GEO Accession GSE67769).

### Comparing the Pleiotropic Effects of Aneuploid Events and Single-Gene Changes in Copy Number

In order to compare the pleiotropic effects of aneuploid events and single-gene changes in copy number we calculated the between-condition variance in relative fitness for each mutation (aneuploid event or single-gene amplification) under the three nutrient-imitated conditions examined. Specifically, for each aneuploid event examined in Fig 3 we determined the between-condition variance in fitness. Next, we performed the same calculation for all single-gene amplifications as determined previously [34]. In this study, Payen et al. determined the fitness effects of single-gene amplifications by pooled competition experiments with a genome-wide collection of yeast ORFs cloned into a low-copy-number (CEN) plasmid [54]. We compared the distribution of fitness differences defined by single-gene changes in copy number to that observed with the aneuploid events examined in Fig 3 (S2A Fig) using an unpaired, two-tailed *t* test.

### Construction of ChrII-Targeted Tamp Pool

In order to create Tamps from deletion collection target strains we constructed two unique CFVs to target the *KanMX* cassettes that replaced Watson and Crick genes, pABS003 and pABS004, respectively. Primers OAS009 and OAS010 were used to amplify the *KanMX* cassette region, which was cloned into the BamHI and EcoRI sites of the CFV YCF4 to produce pABS003. Primers OAS011 and OAS012 were used to amplify the KanMX cassette region, which was cloned into the BamHI and EcoRI sites of the CFV YCF4 to produce pABS004. We then transformed 26 heterozygous yeast deletion target strains with a version of the appropriate CFV linearized with NotI.

Overall, 20 of the 26 heterozygous deletion strains yielded transformants with the expected Tamp. For our subsequent experiments, we chose to pool 21 of the Tamp strains, including the *ybr282wΔ/+* Tamp strain, which also carried an extra copy of chromosome II. We added to this pool the *yal066wΔ/+* heterozygous deletion collection strain to act as a wild-type fitness control; *YAL066W* is a pseudogene. To make the final pool that was used in subsequent competition experiments, the 21 Tamp strains plus the surrogate wild-type control strain were inoculated in minimal media, grown for approximately 12 h at 30°C, the cell densities were normalized, and all 22 strains were pooled together. 2 ml glycerol stocks made with 1 ml 50% glycerol plus 1 ml pooled culture were saved at -80°C.

### Barseq and Fitness Determination for ChrII-Targeted Tamp Pool

To determine the fitness effects of the 21 Tamps in the chrII-targeted pool, we performed chemostat competition experiments with this pool in triplicate under sulfate-, glucose-, and phosphate-limiting conditions. At 5 time points throughout each competition experiment DNA samples were prepared and used to make barseq libraries with the PCR primers OAS013 and OAS014 or OAS029 and OAS030. After purification, the barseq libraries were pooled and loaded onto an Illumina HiSeq. The 6 bp barcode used for multiplexing the samples onto a single lane are indicate in S15 Table. As these reads were obtained from a run that had been multiplexed with other samples unrelated to this study, we have made available tab-delimited files of the raw sequencing data that contain the multiplexing barcode in the first column and the Tamp BC read in the second column. These files can be found at BioProject ID PRJNA257895 with BioSample IDs SAMN02979479 and SAMN02980022 to SAMN029794825. To determine the relative fitness of each of the 21 Tamps in this pool we used an analysis approach that has been successfully used by our lab in a previous publication [34]. Briefly, the frequency of each Tamp at each time point was determined from the barseq reads using a custom pipeline. For each Tamp we then plotted the log_2_(frequency at time = t / frequency at time = 0) versus generations and the slope of the line was taken as the relative fitness. The relative fitness of the *yal066wΔ/+* strain was set at 0 and all the other Tamp fitnesses were normalized to it. The relative fitnesses for all 21 Tamps under all three nutrient-limiting conditions are reported in S5 Table and plotted in S4 Fig. Occasionally, insufficient reads were obtained to calculate the fitness of a particular strain under a particular condition. In this case the fitness is noted as “NA.”

### Construction, Barseq, and Fitness Determination for the ChrII-Targeted Deletion Pool

To develop a method that could confirm the identity of driver genes along a Tamp, we tested a method that paired a single large Tamp with single gene deletions along its length. We generated a MATα 60 kb chrII Tamp strain (chrII-Tamp 1N) as described above and crossed it to 22 MATa deletion strains corresponding to genes within this 60 kb region. These MATa deletion strains were from a minimally passaged collection derived from the yeast magic marker collection [40]. We pooled these 22 strains and competed them in the three nutrient-limiting conditions in triplicate as described for the chrII-targeted Tamp pool. Similarly, we performed barseq on these samples using the same protocol as described for the chrII-targeted Tamp pool. These barseq libraries were pooled together and sequenced on an Illumina HiSeq (the 6 bp barcodes used for multiplexing are reported in S15 Table) and 354,545,894 reads were obtained. As these reads were obtained from a run that had been multiplexed with other samples unrelated to this study, we have made available tab-delimited files of the raw sequencing data that contain the multiplexing barcode in the first column and the Tamp BC read in the second column. These files can be found at BioProject ID PRJNA257895 with BioSample IDs SAMN02979479 and SAMN02980022 to SAMN029794825. Fitnesses were determined for each strain as described for the chrII-targeted Tamp pool, except that they were normalized to the fitnesses of the 60 kb chrII amplification alone (strain “chrII Tamp 2N”) instead of *yal066wΔ/+* and are reported in S5 Table and plotted in S8 Fig.

### Construction of the Genome-Wide Tamp Pool

To construct the genome-wide Tamp pool, 2,254 neutral fitness strains ([34]; S4 Table) from the yeast heterozygous deletion collection (“Magic Marker” collection, [40]) were grown in YPD + G418 (200μg/ml) + 0.18 μg /ml His (+ 50uM riboflavin when recommended) for approximately 24 h at 30°C. We separated these deletion collection strains into two pools depending on the orientation of the KanMX cassette (S3 Fig): **W**atson-strand genes on the **L**eft side of the centromere and **C**rick-strand genes on the **R**ight side of the centromere (wlcr pool) and **W**atson-strand genes on the **R**ight side of the centromere and **C**rick-strand genes on the **L**eft side of the centromere(wrcl pool). We designed two CFVs, one for each pool, that were identical except for the orientation of the KanMX cassette: pAS006 (for the wlcr pool) and pAS007 (for the wrcl pool). In order to maintain a high complexity of the 12 bp replicate BC, approximately 20,000–30,000 *Escherichia coli* colonies transformed with pAS006 and pAS007, respectively, were scraped and used to prepare plasmid DNA (Wizard Miniprep) for yeast transformation.

The wlcr and wrcl yeast heterozygous deletion pools were each transformed with their appropriate CFV. The transformation efficiency with CFVs pABS006 and pABS007 was only about 20% (as determined by a PCR assay), so our pool of scraped colonies included both Tamp strains and original heterozygous deletion strains. However, the design of the PCR primers used to generate our barseq libraries (OAS021 to OAS023) only amplified the strain-identifying barcode from successfully formed Tamp strains. The total number of unique transformants collected was approximately 23,000 and approximately 20,000 for the wlcr and wrcl pools, respectively, and resulted in an average of 26 unique replicates for each Tamp. Given the large number of replicate BCs included in the CFVs pABS006 and pABS007, each transformant was identifiable by a unique combination of the strain-identifying barcode, as derived from the yeast deletion collection barcode (Tamp BC), and the replicate BC (Fig 4A).

To confirm the construction of this pool, we prepared barseq libraries for sequencing using primers OAS021 to OAS023 from aliquots of each pool. These barseq libraries were prepared as described for the chrII-targeted Tamp pool and sequenced on an Illumina MiSeq with sequencing primers OAS024 to OAS027, generating 4,348,080 reads. The fastq files for this barseq experiment are at BioProject ID PRJNA257895 with BioSample IDs SAMN02979480 to SAMN029794821. Additional details about these files are included in S15 Table. Analysis of the barcodes sequenced in this run confirmed that our pool was sufficiently complex to warrant further pooled competition experiments.

As revealed in the construction of our chrII-targeted Tamp pool, generating Tamps using CFVs was not an error-free process and variable karyotypes were sometimes produced. Unfortunately, this problem was exacerbated in the construction of the genome-wide Tamp pool with larger Tamps being more likely to have incorrect karyotypes. The most commonly observed incorrect karyotype was one where the Tamp initiated at the correct genomic location but did not extend all the way to the proximal telomere; this problem was most common for larger Tamps (S7 Table). We adjusted our analysis pipeline to try and correct for these variable karyotypes.

### Pooled Competition Experiments, Barseq, and Fitness Analysis of Genome-Wide Tamp Pool

Similar to the chrII-targeted Tamp competition experiments, we inoculated nine total large volume (approximately 300 ml) nutrient-limited chemostats supplemented with 20 mg/L histidine with aliquots of our wlcr and wrcl pools (both pools were inoculated into a single chemostat). We performed pooled competition experiments under the three different nutrient limited conditions (phosphate-, glucose-, and sulfate-limited) in triplicate; chemostat inoculation and growth were the same as described for the chrII-targeted Tamp pool competition experiments. We defined each of the triplicate chemostat competition experiments as a technical replicate. For each of the nine chemostats, ten time points were taken throughout the competition experiment. For each time point, DNA was extracted and two barseq PCR reactions were carried out (one targeting wlcr Tamps and one targeting wrcl Tamps) using primers OAS021 to OAS023 and resulting in a total of 180 barseq samples. These 180 samples were pooled in equal proportions in two pools of 90 samples each. The pool, 6 bp barcodes used for multiplexing, and generations corresponding to each of the 180 samples are recorded in S15 Table. Each pool was sequenced on three lanes of an Illumina HiSeq, generating a total of 752,336,013 reads. These fastq files are deposited at BioProject ID PRJNA257895 with BioSample IDs SAMN02979482 to SAMN02980021. The method we used to determine the fitness of each Tamp in the pools can be found in S1 Text. The relative fitness for each Tamp and its error are plotted for each condition in S7 Fig.

When we plotted the fitnesses for each Tamp across the genome, we observed that parts of the fitness landscape had a stair-step appearance, in which fitness plateaus were bordered by sharp fitness breakpoints. In order to segment the genome into regions defined by Tamps of similar fitness, we applied the copy-number variant prediction software, DNAcopy [43], to our genome-wide fitness data using the following settings: we required a minimum of two adjacent fitness data to define a fitness plateau and a significance of 0.05 to call a fitness breakpoint. This segmentation defined a total of 250 fitness segments across the three different nutrient-limiting conditions (Colored boxes in S7 Fig).

### Comparing Tamp Fitness Data to Single-Gene Amplification Fitness Data

Previously, our lab determined the fitness effects of single-gene amplifications genome-wide using pooled competition experiments followed by barseq of genome-wide ORF collections on both low-copy-number (CEN) and high-copy-number (2 μ) plasmids [34]. We compared these single-gene amplification data to our genome-wide Tamp data in three ways. First, we compared the kernel density estimates for the fitnesses defined by Tamps to the fitnesses defined by single-gene amplifications (Fig 7A). The kernel density estimates were computed in R. Next, we stratified the 250 groups of Tamps defined by DNAcopy as positive or negative and averaged the fitnesses of all single-gene amplifications contained within its length as determined by their low-copy-number (CEN) fitness effects (S2D Fig). Finally, we examined the breaks between fitness plateaus as defined by our DNAcopy segmentation analysis and categorized each break as either an Upstep (i.e., an increase in fitness moving along the chromosome towards the telomere) or a Downstep (i.e., a decrease in fitness moving along the chromosome towards the telomere). We averaged the fitnesses, as determined by their low-copy-number fitness effects, of all single-gene amplifications contained within each breakpoint region plus one gene centromeric of the centromeric border of the breakpoint region (Fig 7A). This extra gene was included simply to compensate for any insensitivity in the DNAcopy segmentation of our fitness data.

### Identification of Candidate Driver Genes by Comparing Downstep Genes with Previously Published Datasets

As described in the main text, we filtered the list of Downstep genes by comparing it to several published datasets: the list of genes commonly up-regulated in clones evolved under glucose-, phosphate-, or sulfate-limiting conditions [10,46]; the list of genes that increased fitness when present on a low-copy number plasmid under glucose-, phosphate- or sulfate-limiting conditions [34]; and the list of genes mutated in populations evolved under glucose-, phosphate-, or sulfate-limiting conditions [34]. Specifically, for the comparison with the Payen low-copy-number plasmid fitness data, we compared Downstep genes to Payen et al.’s list of outlier fitness genes with fitnesses <-0.10 or >0.10 (denoted as “CEN outlier” in S10 Table and S11 Table) and also to the set of genes with fitnesses greater than two standard deviations more than the mean fitness of that dataset (denoted as “CEN mean + 2SD” in S10 Table and S11 Table). CEN mean + 2SD genes still have extreme fitnesses but did not reach the stringent cutoff imposed in the Payen et al. study to be called “outliers.” For phosphate-limitation this included single-gene amplifications with fitnesses <-0.096 or >0.097, and for glucose-limitation this included single-gene amplifications with fitnesses <-0.052 or >0.050. The list of “outliers” called by Payen et al. already included all mean + 2SD genes for sulfate-limited conditions.

## Supporting Information

S1 Fig Most evolved clones have wild-type growth rates in rich media. Evolved clones, and relevant wild-type controls, were grown in batch culture at 30°C in synthetic complete media. Average doubling times in hours +/- SE are plotted. G3, G4, P3, and P4 clones are FY background and G7, G8, P7, P8, S7, and S8 clones are CEN.PK background. Dashed horizontal lines indicate wild-type doubling times. Clones G8c1, P7c1, and P7c2 have a significantly extended doubling time relative to their appropriate wild type (*p*-values = 0.05, 0.02, and 0.007 respectively, unpaired two-tailed *t* test). Raw data can be found in S16 Table.
(TIFF)

S2 Fig General properties of the Tamps. A) Aneuploid events are more pleiotropic than single gene changes in copy number (unpaired, two-tailed *t* test, *p =* 0.049). The between-condition variance in fitness of single-gene changes in copy number is plotted as a density. CEN = genome-wide collection of yeast strains with each gene cloned into a low-copy-number CEN plasmid **(**raw data from [34], S2 Table **)**. The between-condition variance in fitness of four isolated aneuploid events are plotted on the same *x*-axis, where the color defines the identity of the aneuploid event. Raw data can be found in S2 Table. B) The fitnesses of Tamps, as determined by our pooled competition experiments, agree well with fitnesses determined by head-to-head competition experiments. Adjusted R^2^ = 0.64. Raw data can be found in S8 Table. C) The size of the Tamp does not correlate with its effects on fitness. Adjusted R^2^ = 0.05. Raw data can be found in S6 Table. D) Fitness effects of Tamps cannot be predicted by averaging the fitness effect of all single-gene amplifications along their lengths. Raw data can be found in S9 Table and in S2 Table from [34].
(TIFF)

S3 Fig Generation of Tamps from deletion collection strains. The yeast heterozygous deletion collection allowed us to construct a pool of diverse telomeric amplicon strains using only two CFV designs. The KanMX cassette in deletion collection strains of Watson-strand genes located on the left side of the centromere (A) and Crick-strand genes located on the right side of the centromere (B) (wlcr pool) are in the same orientation relative to the proximal telomere (i.e., the 5′ end of the KanMX cassette is closer to the telomere than the 3′ end of the KanMX) and can be transformed with a single CFV to produce a Tamp. The Tamps are formed via a break-induced-replication (BIR) mechanism initiated at the homology between the KanMX cassette the KanMX fragment cloned into the CFV. Blue boxes represent genomic regions at two copies; pink boxes represent genomic regions at one copy. WT chr = wild-type chromosome, KO chr = chromosome with the gene deletion, CFV = chromosome fragmentation vector.
(TIFF)

S4 Fig Comparison of chrII-targeted Tamp fitnesses under sulfate-, phosphate-, and glucose-limited conditions. Plotted are the mean fitnesses of the 21 Tamps in the chrII-targeted Tamp pool as determined under sulfate- (blue), phosphate- (green), and glucose- (red) limiting conditions. Note that the Tamps are distinguished by the gene at which they initiate and are arranged in genomic order; however, the *x*-axis does not represent their precise spatial distribution along the genome in exact base-pairs. Tamps grown under phosphate- and glucose-limiting conditions generally had neutral or slightly negative fitness effects. Tamps grown under sulfate-limiting conditions that included *SUL1* increased fitness, while those initiating telomeric of the *SUL1* locus did not. The mean fitnesses were all normalized to a pseudogene deletion strain (*YAL066W)* and the error bars represent +/- SE. Raw data can be found in S5 Table.
(TIFF)

S5 Fig Examples of the data used to calculate the relative fitness for each Tamp. This figure shows the data for three biological replicates for the Tamp initiating at *YBR289W* under glucose-, phosphate-, and sulfate-limiting conditions. In total, for the Tamp initiating at *YBR289W*, 100, 82, and 21 biological replicates were tracked under sulfate-, glucose-, and phosphate-limiting conditions, respectively. Each graph represents a single biological replicate as marked by the 12 bp replicate barcode shown above the graph (see Fig 4A). Plotted is the log_2_ ratio of the frequency of the biological replicate at the generation indicated relative to its frequency at generation = 0 over the approximately 20 generations of steady-state competition. Each line, colored black, green, or red, represents one of three technical replicate competition experiments carried out under the indicated condition; some biological replicates were only tracked successfully in one or two out of the three technical replicate experiments. A detailed description of our analysis is provided in S1 Text. Raw data can be found in S18 Table, S19 Table, and S20 Table.
(TIFF)

S6 Fig A comparison of the fitness landscape defined by Tamps and the population frequency of aneuploid events observed in evolution experiments. The fitness of each Tamp as determined under phosphate-, sulfate-, or glucose- limiting conditions is plotted as a vertical bar at the location in the genome where the Tamp initiates (Top half of A, B, and C respectively). For comparison, the population frequency data for the evolved populations containing aneuploid events, from Fig 1A, is aligned beneath the Tamp fitness data (bottom half of A, B, and C for phosphate-, sulfate-, and glucose-limiting conditions, respectively). Raw data can be found in S1 Table and S6 Table.
(TIFF)

S7 Fig Detailed summary of genome-wide Tamp screen. Each point in the following figure represents the fitness determined for the Tamp that initiates at the point in the genome and extends to the proximal telomere. Error bars represent the SE. The dashed line simply connects the fitness data from neighboring Tamp points. The stacked boxes represent Tamps with equivalent fitness as determined by the segmentation program DNAcopy (see S1 Text for analysis details). Boxes enclosing Tamps with fitness >0 are shaded a lighter color than boxes enclosing Tamps with fitness <0. Tamp fitness data as determined under sulfate-, glucose-, and phosphate-limiting conditions are colored blue, red, and green respectively. Raw data can be found in S6 Table.
(TIFF)

S8 Fig Combining a single large Tamp with gene deletions along its length identifies driver genes necessary for the Tamp’s increased fitness. By pairing a large approximately 60 kb Tamp on the right arm of chromosome II with 20 heterozygous gene deletions along its length we can see that *SUL1* is the main driver of fitness under sulfate-limiting conditions (blue) because when its copy number is reduced from three to two, the average fitness decreases from 26% greater than wild type to 2.6% less than wild type. The decrease in copy number from three to two of *SUL1* also appears to decrease fitness under phosphate-limiting conditions; the explanation for this remains unclear. However, the copy-number change of most genes has little effect under glucose- or phosphate-limiting conditions. This method did not identify *BSD2* as a driver of increased fitness under sulfate- or glucose-limiting conditions. Raw data can be found in S5 Table.
(TIFF)

S1 Table Population frequency raw data. (XLSX)

S2 Table Fitnesses determined by direct competition experiments. (XLSX)

S3 Table Illumina sequencing details for sequenced clones and populations used in this study. (XLSX)

S4 Table Heterozygous gene deletions with neutral fitness under glucose-, sulfate-, and phosphate-limiting conditions as reported in [34]. (XLSX)

S5 Table Raw data for chrII targeted pool: Tamp pool and large Tamp + single-gene deletion pool. (XLSX)

S6 Table Tamp fitnesses as predicted by genome-wide screen. (XLSX)

S7 Table Karyotype verification of clones isolated from Tamp pool. (XLSX)

S8 Table Fitness verification of clones isolated from Tamp pool. (XLSX)

S9 Table Regions of equivalent fitness as predicted by the segmentation program DNAcopy. (XLSX)

S10 Table Detailed analysis of Tamps identified from evolution experiments. (XLSX)

S11 Table Candidate driver genes. (XLSX)

S12 Table Strains used in this study. (XLSX)

S13 Table Plasmids used in this study. (XLSX)

S14 Table Primers used in this study. (XLSX)

S15 Table Illumina multiplexing barcodes for all barseq samples. (XLSX)

S16 Table Doubling time (in hours) for strains examined in this study. (XLSX)

S17 Table Breakpoint classification and breakpoint genes used for analysis. (XLSX)

S18 Table Raw data for Tamp competition carried out under glucose limitation. This. csv file contains the following columns: the gene at which the Tamp initiates (“gene”), the biological replicate as indicated by the unique 10 bp barcode (“mer”), the technical replicate (“replicate”), the log_2_ ratio of the frequency of the Tamp relative to time point t = 0 after adding “1” to all read counts (t1–t9), the generations elapsed for time points 1–9 (g1 to g9).
(TXT)

S19 Table Raw data for Tamp competition carried out under phosphate limitation. This. csv file contains columns identical to those of S18 Table.
(TXT)

S20 Table Raw data for Tamp competition carried out under sulfate limitation. This. csv file contains columns identical to those of S18 Table.
(TXT)

S1 Text Supplementary methods. (DOCX)

## Abbreviations

aCGH: array comparative genomic hybridization
BIR: break-induced-replication
CFV: chromosome fragmentation vector
CNV: copy-number variant
GFP: green fluorescent protein
GO: Gene Ontology
SNVs: single-nucleotide variants
Tamp: telomeric amplicon
WGS: whole-genome sequencing
