# Supplementary figures and images for: The Fitness Consequences of Aneuploidy Are Driven by Condition-Dependent Gene Effects
Source: PLoS Biol. 2015 May 26;13(5):e1002155. doi: 10.1371/journal.pbio.1002155 (PMC4444335; doi:10.1371/journal.pbio.1002155)

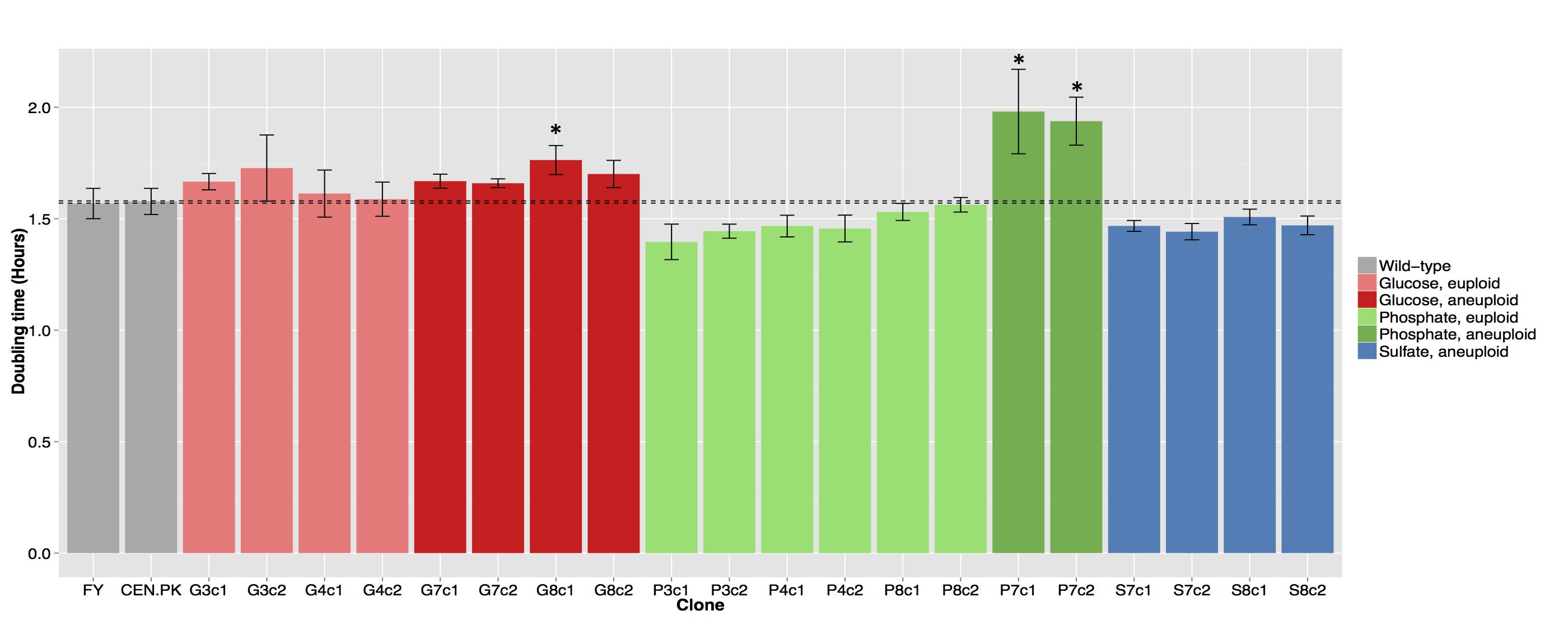

Supplement: S1 Fig — Evolved clones, and relevant wild-type controls, were grown in batch culture at 30°C in synthetic complete media. Average doubling times in hours +/- SE are plotted. G3, G4, P3, and P4 clones are FY background and G7, G8, P7, P8, S7, and S8 clones are CEN.PK background. Dashed horizontal lines indicate wild-type doubling times. Clones G8c1, P7c1, and P7c2 have a significantly extended doubling time relative to their appropriate wild type (p-values = 0.05, 0.02, and 0.007 respectively, unpaired two-tailed t test). Raw data can be found in S16 Table. (TIFF) [file pbio.1002155.s001.tiff]

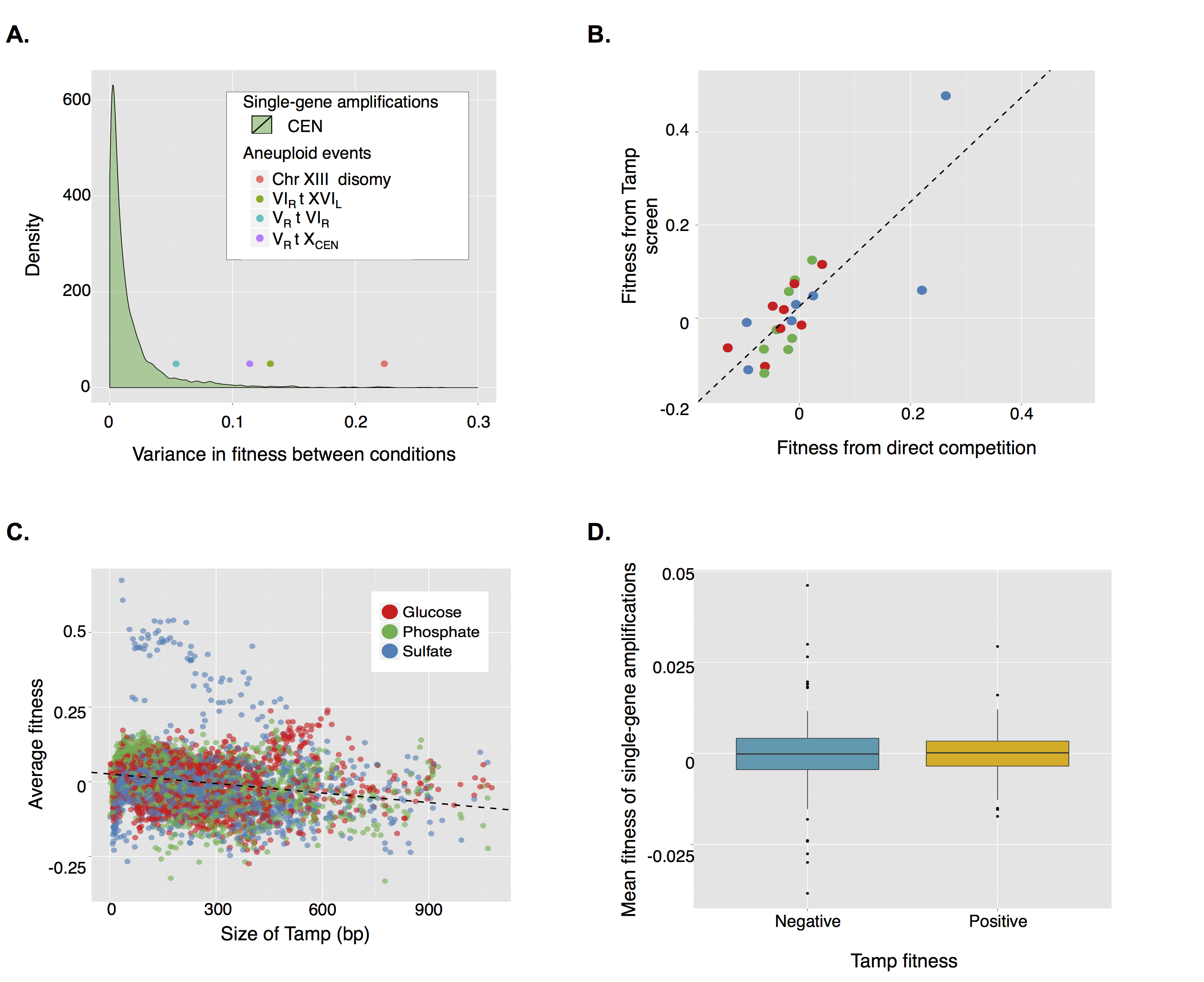

Supplement: S2 Fig — A) Aneuploid events are more pleiotropic than single gene changes in copy number (unpaired, two-tailed t test, p = 0.049). The between-condition variance in fitness of single-gene changes in copy number is plotted as a density. CEN = genome-wide collection of yeast strains with each gene cloned into a low-copy-number CEN plasmid (raw data from [34], S2 Table ). The between-condition variance in fitness of four isolated aneuploid events are plotted on the same x-axis, where the color defines the identity of the aneuploid event. Raw data can be found in S2 Table. B) The fitnesses of Tamps, as determined by our pooled competition experiments, agree well with fitnesses determined by head-to-head competition experiments. Adjusted R2 = 0.64. Raw data can be found in S8 Table. C) The size of the Tamp does not correlate with its effects on fitness. Adjusted R2 = 0.05. Raw data can be found in S6 Table. D) Fitness effects of Tamps cannot be predicted by averaging the fitness effect of all single-gene amplifications along their lengths. Raw data can be found in S9 Table and in S2 Table from [34]. (TIFF) [file pbio.1002155.s002.tiff]

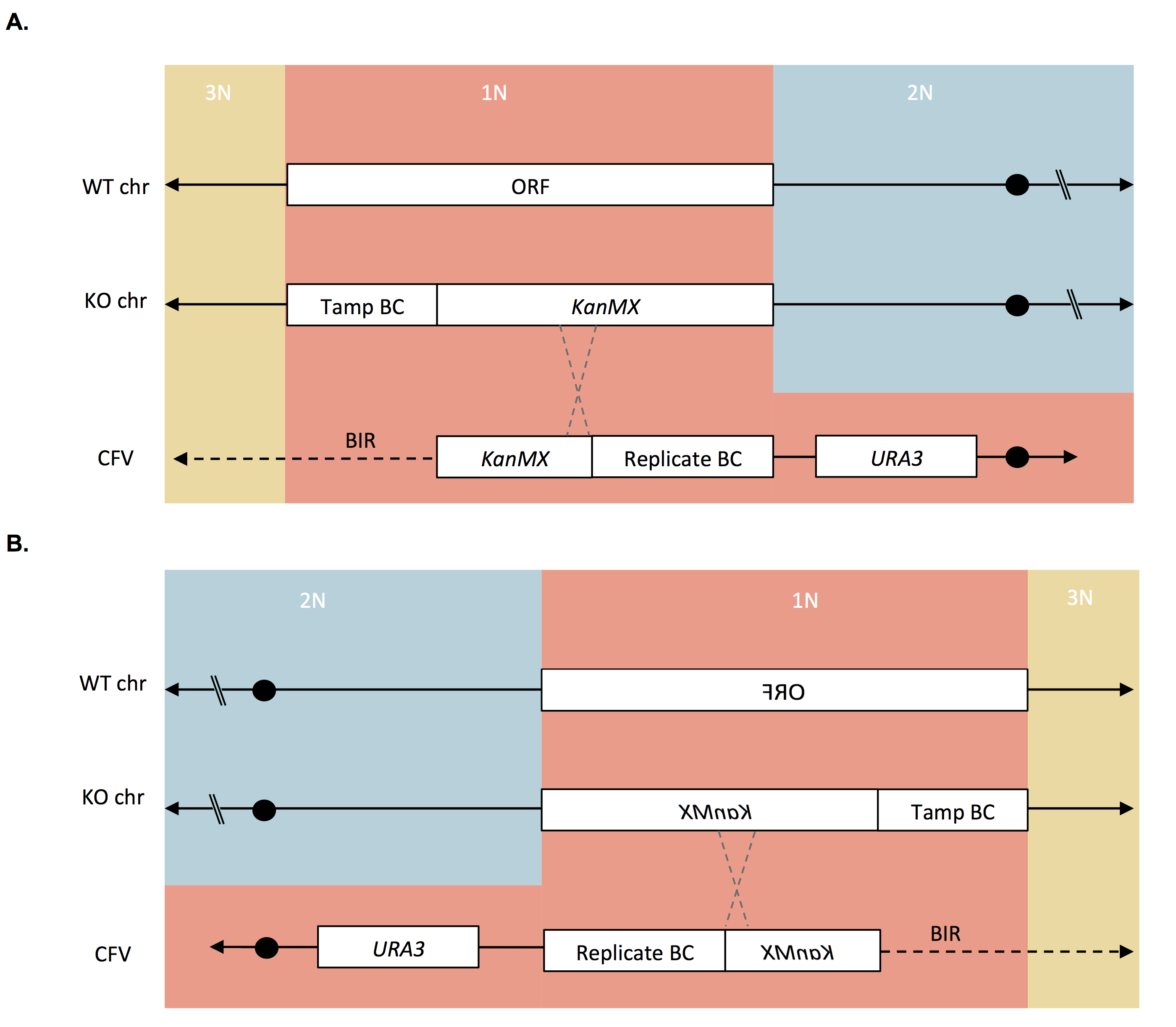

Supplement: S3 Fig — The yeast heterozygous deletion collection allowed us to construct a pool of diverse telomeric amplicon strains using only two CFV designs. The KanMX cassette in deletion collection strains of Watson-strand genes located on the left side of the centromere (A) and Crick-strand genes located on the right side of the centromere (B) (wlcr pool) are in the same orientation relative to the proximal telomere (i.e., the 5′ end of the KanMX cassette is closer to the telomere than the 3′ end of the KanMX) and can be transformed with a single CFV to produce a Tamp. The Tamps are formed via a break-induced-replication (BIR) mechanism initiated at the homology between the KanMX cassette the KanMX fragment cloned into the CFV. Blue boxes represent genomic regions at two copies; pink boxes represent genomic regions at one copy. WT chr = wild-type chromosome, KO chr = chromosome with the gene deletion, CFV = chromosome fragmentation vector. (TIFF) [file pbio.1002155.s003.tiff]

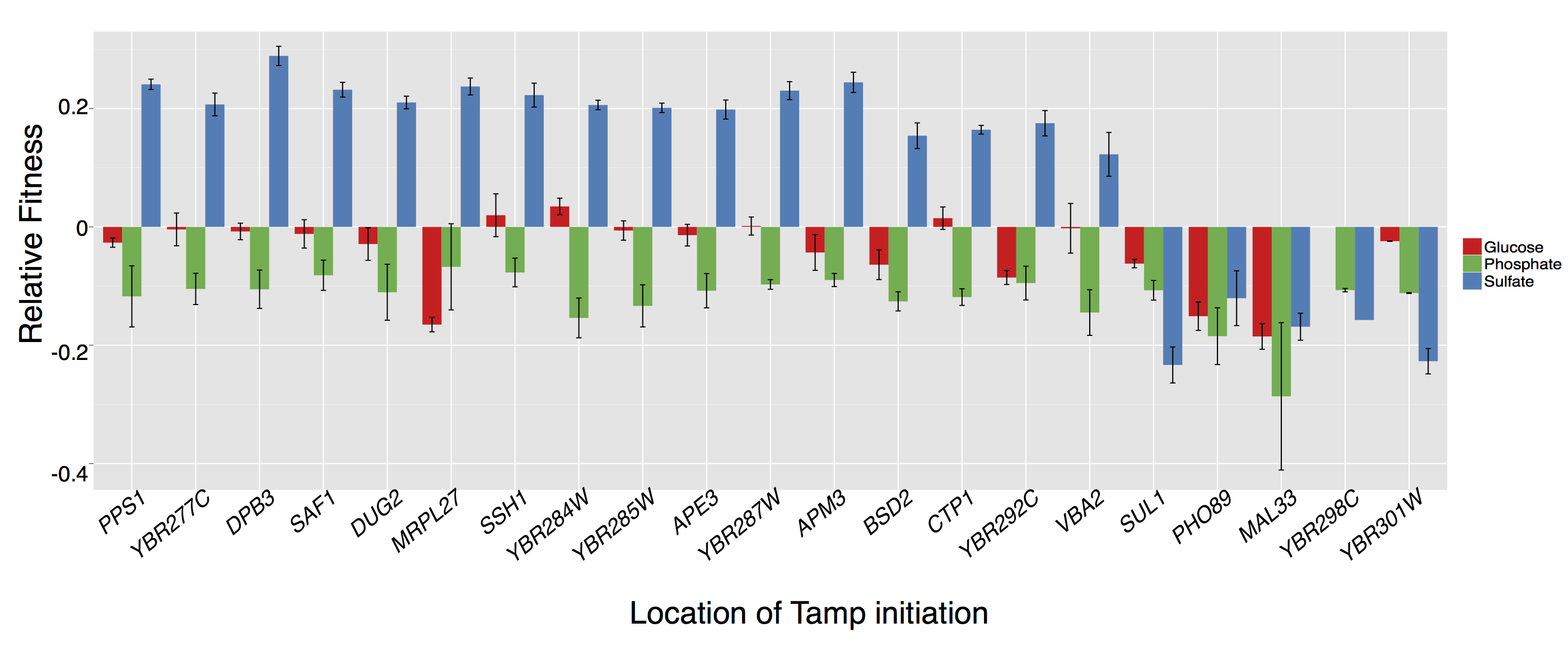

Supplement: S4 Fig — Plotted are the mean fitnesses of the 21 Tamps in the chrII-targeted Tamp pool as determined under sulfate- (blue), phosphate- (green), and glucose- (red) limiting conditions. Note that the Tamps are distinguished by the gene at which they initiate and are arranged in genomic order; however, the x-axis does not represent their precise spatial distribution along the genome in exact base-pairs. Tamps grown under phosphate- and glucose-limiting conditions generally had neutral or slightly negative fitness effects. Tamps grown under sulfate-limiting conditions that included SUL1 increased fitness, while those initiating telomeric of the SUL1 locus did not. The mean fitnesses were all normalized to a pseudogene deletion strain (YAL066W) and the error bars represent +/- SE. Raw data can be found in S5 Table. (TIFF) [file pbio.1002155.s004.tiff]

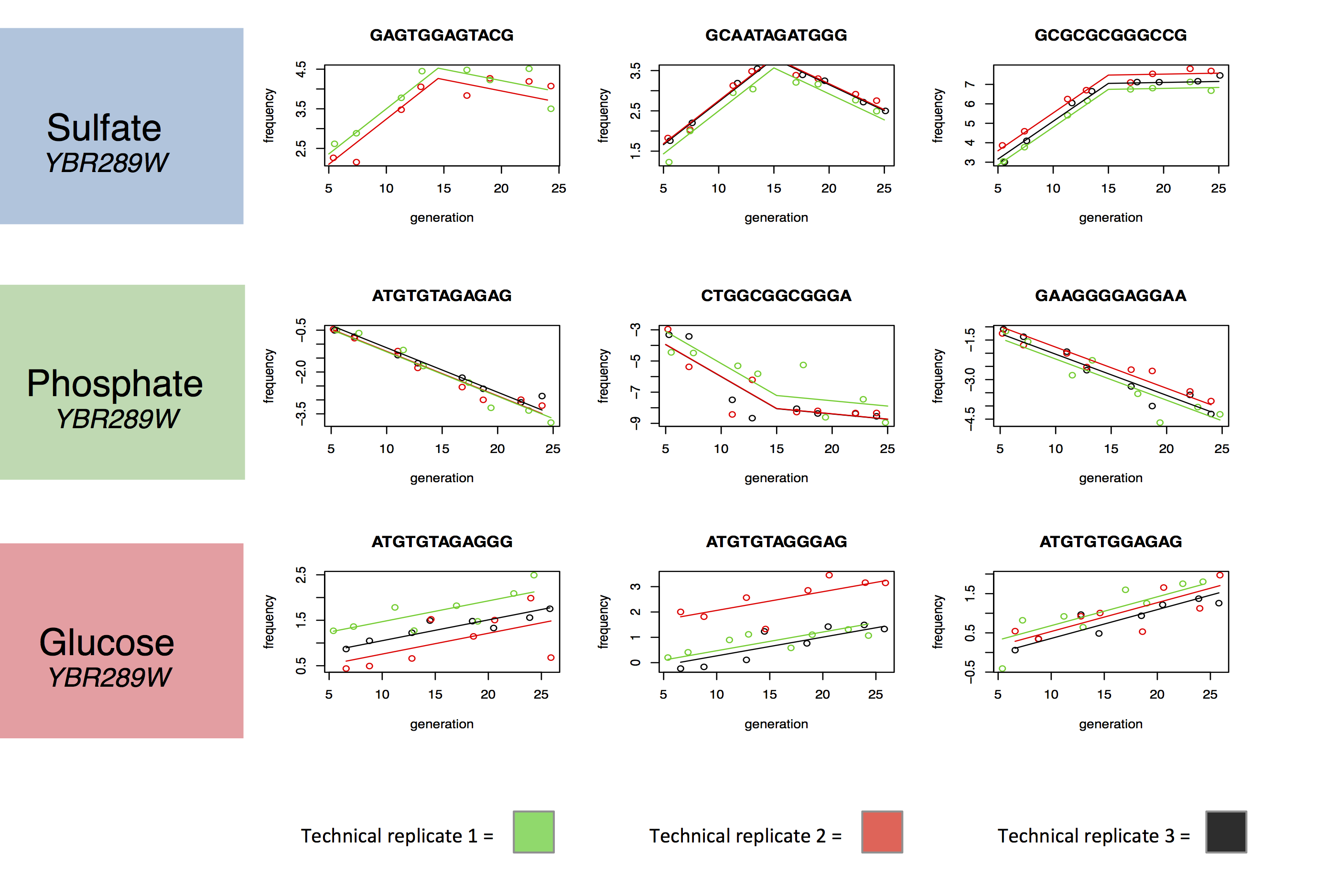

Supplement: S5 Fig — This figure shows the data for three biological replicates for the Tamp initiating at YBR289W under glucose-, phosphate-, and sulfate-limiting conditions. In total, for the Tamp initiating at YBR289W, 100, 82, and 21 biological replicates were tracked under sulfate-, glucose-, and phosphate-limiting conditions, respectively. Each graph represents a single biological replicate as marked by the 12 bp replicate barcode shown above the graph (see Fig 4A). Plotted is the log2 ratio of the frequency of the biological replicate at the generation indicated relative to its frequency at generation = 0 over the approximately 20 generations of steady-state competition. Each line, colored black, green, or red, represents one of three technical replicate competition experiments carried out under the indicated condition; some biological replicates were only tracked successfully in one or two out of the three technical replicate experiments. A detailed description of our analysis is provided in S1 Text. Raw data can be found in S18 Table, S19 Table, and S20 Table. (TIFF) [file pbio.1002155.s005.tiff]

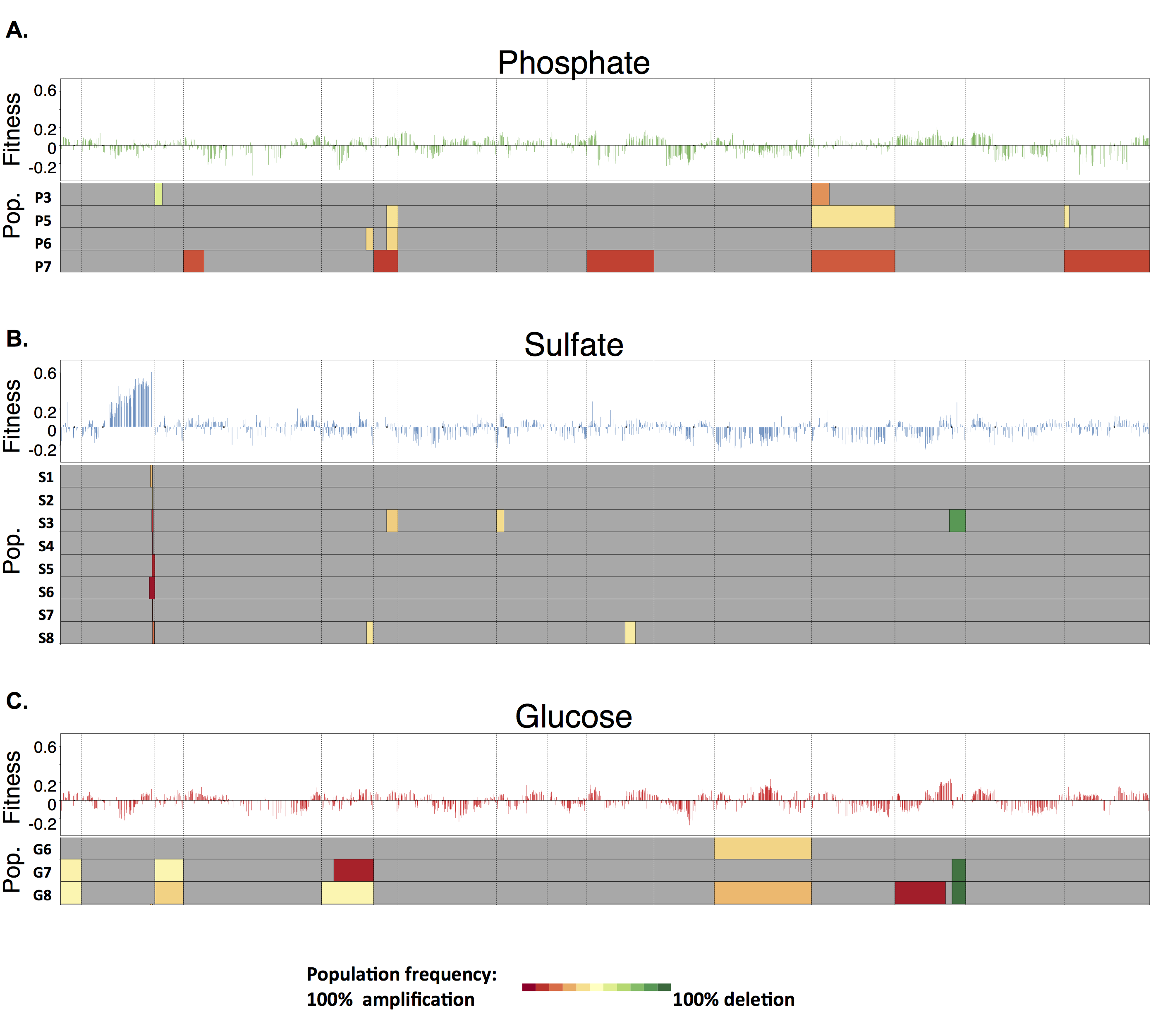

Supplement: S6 Fig — The fitness of each Tamp as determined under phosphate-, sulfate-, or glucose- limiting conditions is plotted as a vertical bar at the location in the genome where the Tamp initiates (Top half of A, B, and C respectively). For comparison, the population frequency data for the evolved populations containing aneuploid events, from Fig 1A, is aligned beneath the Tamp fitness data (bottom half of A, B, and C for phosphate-, sulfate-, and glucose-limiting conditions, respectively). Raw data can be found in S1 Table and S6 Table. (TIFF) [file pbio.1002155.s006.tiff]

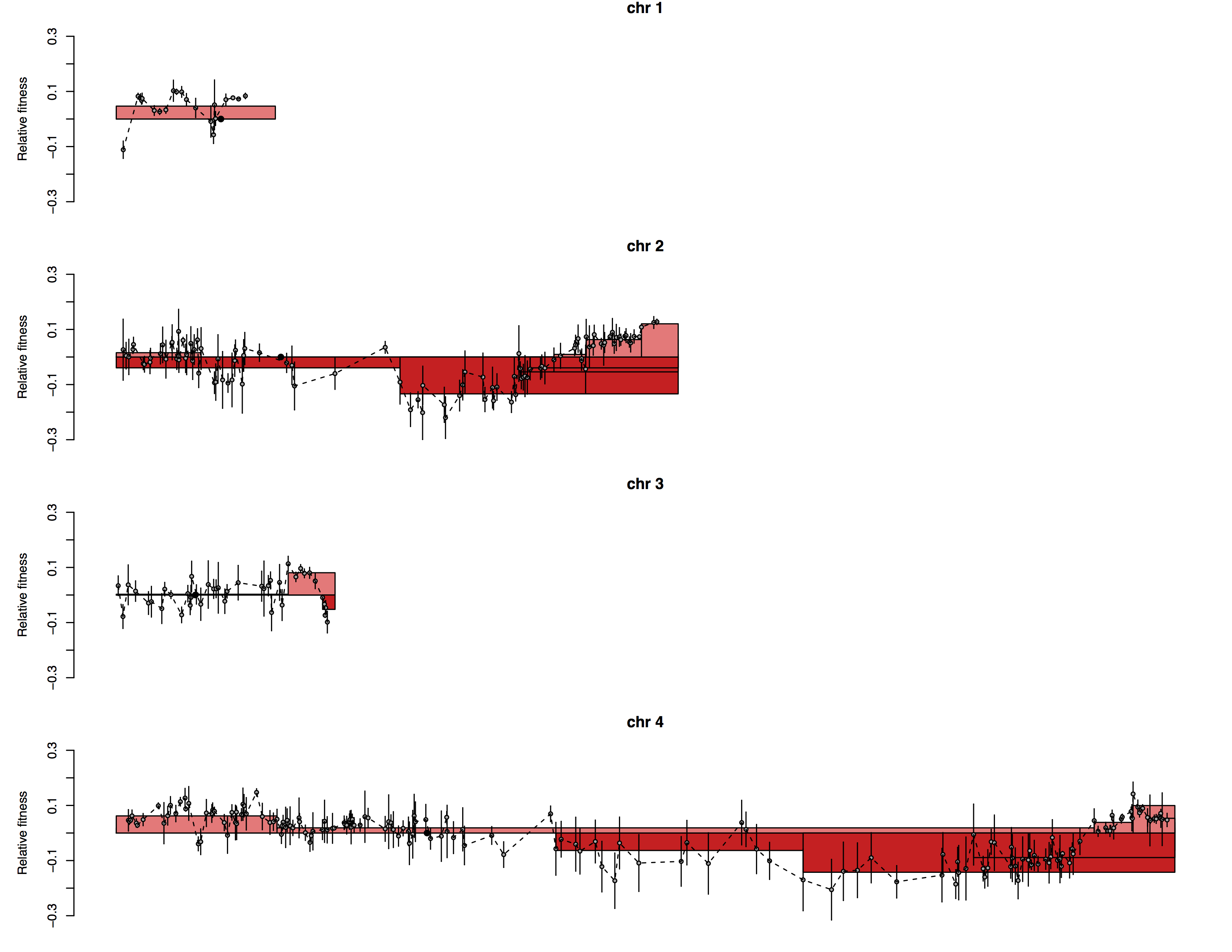

Supplement: S7 Fig — Each point in the following figure represents the fitness determined for the Tamp that initiates at the point in the genome and extends to the proximal telomere. Error bars represent the SE. The dashed line simply connects the fitness data from neighboring Tamp points. The stacked boxes represent Tamps with equivalent fitness as determined by the segmentation program DNAcopy (see S1 Text for analysis details). Boxes enclosing Tamps with fitness >0 are shaded a lighter color than boxes enclosing Tamps with fitness <0. Tamp fitness data as determined under sulfate-, glucose-, and phosphate-limiting conditions are colored blue, red, and green respectively. Raw data can be found in S6 Table. (TIFF) [file pbio.1002155.s007.tiff]

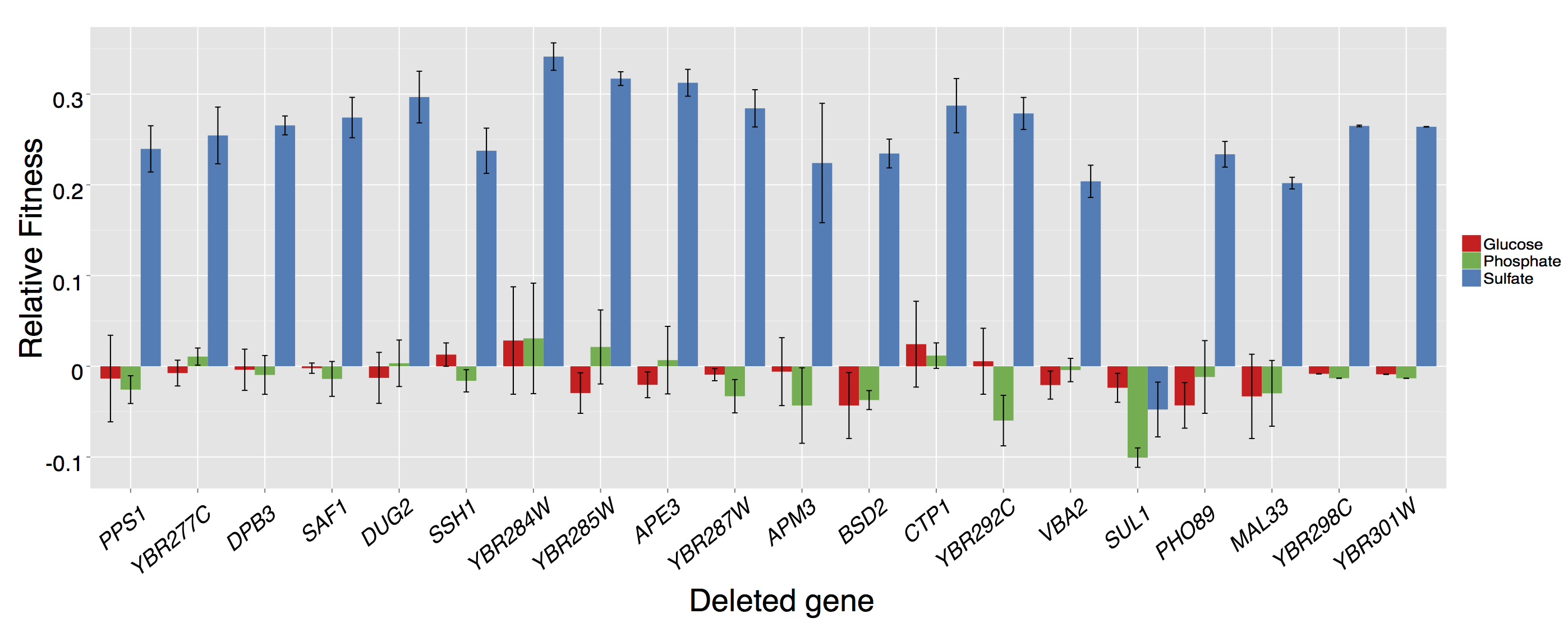

Supplement: S8 Fig — By pairing a large approximately 60 kb Tamp on the right arm of chromosome II with 20 heterozygous gene deletions along its length we can see that SUL1 is the main driver of fitness under sulfate-limiting conditions (blue) because when its copy number is reduced from three to two, the average fitness decreases from 26% greater than wild type to 2.6% less than wild type. The decrease in copy number from three to two of SUL1 also appears to decrease fitness under phosphate-limiting conditions; the explanation for this remains unclear. However, the copy-number change of most genes has little effect under glucose- or phosphate-limiting conditions. This method did not identify BSD2 as a driver of increased fitness under sulfate- or glucose-limiting conditions. Raw data can be found in S5 Table. (TIFF) [file pbio.1002155.s008.tiff]
