# Supplementary material for: The Fitness Consequences of Aneuploidy Are Driven by Condition-Dependent Gene Effects
Source: PLoS Biol. 2015 May 26;13(5):e1002155. doi: 10.1371/journal.pbio.1002155 (PMC4444335; doi:10.1371/journal.pbio.1002155)
Supplement: S1 Text — (DOCX) [file pbio.1002155.s029.docx]

**S1 Text**

**Supplemental Materials and Methods:**

Strains, media and primers

The strains, plasmids and primers used in this study are listed in **S13 Table, S14 Table,** and **S15 Table** respectively. Unless specified below, yeast strains were grown at 30°C and standard media recipes were used.

aCGH to determine population frequency of aneuploid events in evolved populations

Previously, 24 evolution experiments were carried out under nutrient-limiting conditions [1]**.** At that time population DNA was isolated at or near the final timepoint from five of the evolution experiments. aCGH of those population DNA samples was used to determine the frequency of the aneuploid events in those populations. See [1] **S3 Fig.** for additional details. In order to determine the population frequency of the aneuploid events present in the remaining evolution experiments, we generated population DNA from archived glycerol stocks saved from the final timepoint or near the final timepoint of the remaining 19 evolution experiments. We made population DNA from these samples by resuspending a small amount of each glycerol stock in YPD, plating ~1000 colonies to YPD plates, scraping colonies and extracting DNA with a modified Hoffman-Winston protocol (See <http://dunham.gs.washington.edu/UWyeastDNAprep.pdf> for additional details). This population DNA was labeled and hybridized to Agilent 4x44K microarrays as described in http://dunham.gs.washington.edu/UWAgilentDNA.pdf. Population frequencies of aneuploid events observable by aCGH were determined by comparing their known or, in the case of the *SUL1* amplicons, inferred clonal copy number to the copy number determined by aCGH of the population DNA. All aCGH data are available at GEO Accession GSE67769. As we wanted to ensure that the population frequencies determined by this method accurately represented the population frequencies present in the original evolution experiment population, we compared the population frequency estimates for population P7 as determined previously in [1] from fresh population DNA samples to the frequencies determined by the method described here and found similar results (the following list describes each aneuploid chromosome’s population frequency in the following format: chr = population frequency as calculated in this study vs. population frequency as calculated in [1]): chrVI = 0.51 vs. 0.69; chrVI = 0.92 vs. 0.77; chrX = 0.58 vs. 0.77; chrXIII = 0.44 vs. 0.66; chrXVI = 0.68 vs. 0.73. As described in the main text, this approach was additionally confirmed by a PCR assay that amplified the breakpoint of the V_R_ t X_CEN_ translocation event present in population S8 using primers OAS005-0AS0008. This breakpoint PCR assay identified the V_R_ t X_CEN_ supernumerary chromosome in 13 of 98 total clones tested (13%). Our population aCGH determined the frequency of the chromosome V_R_ amplification to be 15% and the chromosome X_CEN_ to be 11% and therefore we predicted the population frequency of the V_R_ t X_CEN_ supernumerary chromosome to be 13%.

Chemostat competition experiments to determine relative fitness

To determine relative fitness, we competed individual clones of test strains against an appropriate control strain with eGFP integrated at the HO locus in nutrient limited chemostats. We used both large volume (~300ml) and small volume (20ml) [2] chemostats for competition experiments. A single colony of each control or test strain was used to start an overnight culture in the same media in which the competition experiment was to be carried out; the overnight culture was then grown at 30°C for ~12-36 hours. 1ml of this overnight was used to inoculate each chemostat which was then allowed to grow at 30°C without dilution for ~30 hours at which point fresh media was added to the culture chamber at a rate of 0.17 h^-1^. After achieving steady-state, 50% of a control-strain chemostat was mixed with 50% of a test-strain chemostat resulting in two chemostat replicates for a single competition experiment. Flow-cytometery using a BD Accuri C6 flow cytometer (BD Biosciences) at regular intervals throughout the competition allowed us to track the percentage of GFP-marked control cells over time. The data were plotted with ln[(dark cells/GFP+ cells)] vs. generations and we defined the slope of this relationship as the relative fitness of the test strain. The number replicate competition experiments as well as the appropriate control strain is detailed for all test strains in **S2** **Table**.

Construction of targeted Tamps by individual transformation

Two Tamp strains were constructed individually by direct transformation with a chromosome-fragmentation vector (CFV). 250bp of homology to the genomic location at which we desired to create a Tamp was cloned into the multiple cloning site of the previously designed CFV YCF4 [3]. To create a 78kb Tamp on the right arm of chromosome V (chrVR-Tamp), 250bp of chromosome V was amplified with primers OAS001 and OAS002, cloned into the EcoRI and BamHI sites of YCF4 creating pABS002 which was the transformed into a *MAT*α *ura3-52* FY background strain using a modified Li-Ac protocol. The karyotype was confirmed by aCGH (see GEO Accession GSE67769). This strain was backcrossed to a mata *ura3-52* FY background strain to create a diploid (chrVR-Tamp 2N). To create a 60kb Tamp on the right arm of chrII (chrIIR-Tamp), 250bp of chrII was amplified with primers OAS003 and OAS004, cloned into the EcoRI and BamHI sites of YCF4 creating pABS005 which was the transformed into a *MAT*α *ura3-52 trp1∆63* FY background strain using a modified Li-Ac protocol. The karyotype was confirmed by aCGH (see GEO Accession GSE67769). This strain was backcrossed to a *MATa* *ura3-52* FY background strain to create a diploid (chrIIR-Tamp 2N).

Illumina sequencing of evolved clones and populations

DNA samples from clones isolated from evolution experiments as well as population DNA, generated as described for the population frequency calculations determined by aCGH, were prepared for WGS using Illumina Nextera kits according to the provided protocol. Libraries were sequenced on either an Illumina HiSeq or a GAII generating the number of reads detailed in **S3** **Table**. Reads were aligned with BWA [4] and SNVs were called using the samtools [5] mpileup command after applying standard filters. Specifically, non-uniquely mapping reads, reads in which the pair did not map, reads with a mapping quality less than 30 and PCR/optical duplicate reads were filtered out; the samtools C-50 filter was applied as recommended for reads mapped with BWA and the vcfutils.pl varFilter -D filter was applied with D set to 2X the average mapping coverage. SNVs unique to the evolved clones were identified with a custom Python script, annotated with a second Python script (http://depts.washington.edu/sfields/software/annotate/) [6] and further prioritized by manual examination with the Integrative Genome Viewer (IGV) [7]. Population frequency of SNVs was determined from the allele frequency displayed in IGV. The clones and populations analyzed here (P6c1, P6, P5c3, P5, S8c2 and S8) were included in a previous analysis [8] and the raw data is deposited at BioProject ID PRJNA248591 and BioSample numbers SAMN02800460 (S8c2), SAMN02800438 (P6c1), SAMN02800436 (P5c3, run 1), SAMN02800435 (P5c3, run 2), SAMN02800403 CEN.PK WT diploid, run 1) and SAMN02800404 (CEN.PK WT diploid, run 2).

Backcrossing to isolate aneuploid events and SNVs from evolved clones into wild-type background

To isolate individual mutations (both SNVs and aneuploid events) identified by WGS of the evolved clones P6c1, P5c3, we backcrossed each evolved clone to an isogenic wild-type strain of the opposite mating type, sporulated, and tetrads dissected using standard sporulation media and protocols. Evolved clone S8c2, a diploid, was itself sporulated and tetrads dissected using standard sporulation media and protocols. After Sanger-sequenced confirmed the SNVs identified by WGS, tetrads were genotyped by Sanger sequencing and backcrossed repeatedly until each SNV and aneuploid event was isolated into an otherwise wild-type background. Spores isolated from S8c2 with the desired genotype were backcrossed a final time so that each mutation was once again in a diploid background. The karyotypes were confirmed by aCGH for most clones eventually used for relative fitness competition experiments (see GEO Accession GSE67769).

Comparing the pleiotropic effects of aneuploid events and single-gene changes in copy number

To compare the pleiotropic effects of aneuploid events and single-gene changes in copy number we calculated the between-condition variance in relative fitness for each mutation (aneuploid event or single-gene amplification) under the three nutrient-imitated conditions examined. Specifically, for each aneuploid event examined in **Fig. 3** we determined the between-condition variance in fitness. Next, we performed the same calculation for all single-gene amplifications as determined previously [8]. In this study Payen et al. determined the fitness effects of single-gene amplifications by pooled competition experiments with a genome-wide collection of yeast ORFs cloned into a low-copy-number (CEN) plasmids [9]. We compared the distribution of fitness differences defined by single-gene changes in copy number to that observed with the aneuploid events examined in **Fig. 3** (**S2A** **Fig.**) using an unpaired, two-tailed t-test.

Construction of chrII-targeted Tamp pool

We attempted to generate 26 unique telomeric amplicon strains with Tamps initiating at the locations of 26 different genes within this 60 kb region: targeting genes *YBR276C* to *YBR301W*. Due to the mechanism by which CFV transformation generates Tamps, via a break-induced replication process (BIR) [10], we constructed two unique CFVs to target the *KanMX* cassettes that replaced Watson and Crick genes, pABS003 and pABS004, respectively. This was necessary as the *KanMX* cassette is in reverse orientation with respect to the centromere and proximal telomere for deleted Watson and Crick genes. Primers OAS009 and OAS010 were used to amplify the *KanMX* cassette region which was cloned into the BamHI and EcoRI sites of the CFV YCF4 to produce pABS003. Primers OAS011 and OAS012 were used to amplify the KanMX cassette region which was cloned into the BamHI and EcoRI sites of the CFV YCF4 to produce pABS004. We then transformed each of the chosen 26 heterozygous yeast deletion strains with a linearized, via NotI digestion, version of the appropriate CFV.

Overall, 20 of the 26 heterozygous deletion strains yielded transformants with the expected karyotype. First, using a breakpoint PCR assay that amplified the novel junction created, we determined that 21% of all transformant colonies likely had the desired Tamp. But as we were concerned that CFV transformation might generate off-target aneuploid events, for each PCR-confirmed Tamp strain we then determined the full karyotype using aCGH (see GEO Accession GSE67769). Of 25 clones from 23 target strains we identified as probable transformants by our PCR assay, 20 of them had the appropriate karyotype as confirmed by aCGH and this low frequency of off target effects suggested this method was effective in creating targeted Tamps. An additional three strains yielded transformants with large, off-target aneuploid events. Specifically, the *YBR289WΔ/+* target strain appeared to have triploidized in the original deletion collection, the *YBR282WΔ/+* target strain contained an entire extra copy of chromosome II in the original deletion collection and the *YBR295WΔ/+* strain generated transformants with irreproducible and complex karyotypes. An additional two of the 20 successful transformants contained deletions of the copy-number polymorphic *ASP3* and *CUP1* gene clusters but were still included in our pool competition experiment. For our subsequent experiments, we chose to pool 21 of the telomeric amplicon strains, including the two strains with the gene cluster deletions as well as the *YBR282WΔ/+* strain carrying the extra copy of chromosome II. We added to this pool the *YAL066WΔ/+* heterozygous deletion collection strain to act as a wild-type fitness control: *YAL066W* is a psuedogene. All 21 Tamp strains plus the surrogate wild-type control strain were used to inoculate overnight cultures in minimal media. After ~12 hours of growth at 30°C, the cell densities were normalized and all 22 strains were pooled together. 2ml glycerol stocks made with 1ml 50% glycerol plus 1ml pooled culture were saved at -80°C.

Barseq and fitness determination for chrII-targeted Tamp pool

To determine the fitness effects of the 21 Tamps in the chrII-targeted pool, we performed chemostat competition experiments with this pool under sulfate-, glucose-, and phosphate-limiting conditions. A large-volume, ~300ml, nutrient-limited chemostat was inoculated with a single 2ml glycerol stock sample of our 21 Tamp pool that had been thawed and resuspended in the appropriate media. After allowing the chemostat to grow at 30°C without dilution for ~24 hours, fresh media was added to the chemostat at a rate of 0.17 hour^-1^. This pooled competition experiment was repeated 3 times for each nutrient limitation; these replicates are considered technical replicates. At 5 timepoints throughout each competition experiment cells were sampled and DNA extracted using the modified Hoffman-Winston prep referenced above. PCR using primers OAS013 and OAS014 were used to amplify a 140bp product targeting the deletion collection Uptag located in each strain. Except for the replicate 3 under sulfate-limition for which PCR was performed using primers OAS029 and OAS030 to amplify a 140bp product targeting the deletion collection Downtag located in each strain. After purification with Zymo columns (Zymo Research, D4005), equal ng of each sample were pooled and the pool was purified using the AMPure purification system (Agencourt, A63880). We checked the size and successful purification of primers from our pool by running a small aliquot on a 6% acrylamide gel (Life Technologies EC6265BOX). Our final barseq libraries were pooled and loaded onto an Illumina HiSeq. The 6bp barcode used for multiplexing the samples onto a single lane are indicate in **S15 Table**. As these reads were obtained from a run that had been multiplexed with other samples unrelated to this study, we have made available tab-delimited files of the raw sequencing data that contain the multiplexing barcode in the first column and the Tamp BC read in the second column. These files can be found at BioProject ID PRJNA257895 with BioSample IDs SAMN02979479 and SAMN02980022 to SAMN029794825.

To determine the relative fitness of each of the 21 Tamps in this pool we used an analysis approach that has been successfully used by our lab in a previous publication [8]. Briefly, the frequency of each Tamp at each timepoint was determined from the barseq reads using a custom pipeline. For each Tamp we then plotted the log_2_(frequency at time = t / frequency at time = 0) vs. generations and the slope of the line was taken as the relative fitness. The relative fitness of the *YAL066W +/-* strain was set at 0 and all the other Tamp fitnesses were normalized to it. The relative fitnesses for all 21 Tamps under all three nutrient-limiting conditions are reported in **S5** **Table** and plotted in **S4** **Fig**. Occasionally, insufficient reads were obtained to calculate the fitness of a particular strain under a particular condition. In this case the fitness is noted as ‘NA’.

Construction, barseq and fitness determination for the chrII-targeted deletion pool

To develop a method that could confirm the identity of driver genes along a Tamp, we tested a method that paired a single large Tamp with single gene deletions along its length. We generated a MATα 60kb chrII Tamp strain (chrII-Tamp 1N) as described above and crossed it to 22 MATa deletion strains corresponding to genes within this 60kb region. These MATa deletion strains were from a minimally passaged collection derived from the yeast magic marker collection[11]. We pooled these 22 strains and competed them in under the three nutrient-limiting conditions in triplicate as described for the chrII-targeted Tamp pool. Similarly, we performed barseq on these samples using the same protocol as described for the chrII-targeted Tamp pool. These barseq libraries were pooled together and sequenced on an Illumina HiSeq (the 6bp barcodes used for multiplexing are reported in **S15 Table**) and 354,545,894 reads were obtained. As these reads were obtained from a run that had been multiplexed with other samples unrelated to this study, we have made available tab-delimited files of the raw sequencing data that contain the multiplexing barcode in the first column and the Tamp BC read in the second column. These files can be found at BioProject ID PRJNA257895 with BioSample IDs SAMN02979479 and SAMN02980022 to SAMN029794825. Fitnesses were determined for each strain as described for the chrII-targeted Tamp pool except that they were normalized to the fitnesses of the 60 kb chrII amplification alone (strain ‘chrII Tamp 2N’) instead of *yal066wΔ/+* and are reported in **S5** **Table** and plotted in **S8** **Fig**. As the *YBR289WΔ* strain was derived from a *YBR289WΔ/+* strain that appeared to have triploidized in the original deletion collection, we decided to exclude the chrII Tamp + *YBR289WΔ/+* strain from subsequent analysis.

Construction of the genome-wide Tamp pool

Construction of the genome-wide Tamp pool was very similar to construction of the chrII-targeted Tamp pool. First, 2,254 neutral fitness ([8]; **S4** **Table**) strains from the yeast heterozygous deletion collection (‘Magic Marker’ collection, [11]) were grown in YPD + G418 (200μg/ml) + 0.18 μg /ml His (+ 50uM riboflavin when recommended) for ~24 hours at 30°C. We separated these deletion collection strains into two pools depending on depending on the orientation of the KanMX cassette (**S3** **Fig.**). 1,122 deletion strains of **W**atson-strand genes on the **L**eft side of the centromere and **C**rick-strand genes on the **R**ight side of the centromere (wlcr pool) were pooled together and 1,132 deletion strains of **C**rick-strand genes on the **L**eft side of the centromere and **W**atson-strand genes on the **R**ight side of the centromere (wrcl pool) were combined in a second pool. 2 ml glycerol stock aliquots were saved from each of these pools: 1ml pool plus 1ml 50% glycerol. These aliquots were stored at -80°C.

As described for the construction of the chrII-targeted pool, we needed to separate the wlcr and wrcl gene deletion target strains because the KanMX cassettes in the two pools are in reverse orientation with respect to the centromere and proximal telomere. We therefore also needed to design two CFVs, one for each pool, that were identical except for the orientation of the KanMX cassette; transformation with the wrong CFV would lead to a dicentric chromosome produced via BIR and result in poor viability and variable karyotypic outcomes [10]. To construct the two CFVs we used a nested PCR to amplify two 250bp regions from the KanMX cassette using primers OAS017, OAS018 and OAS020 (for the wlcr CFV) or primers OAS0178 OAS019 and OAS020 (for the wrcl CFV). Primer OAS018 contained 12bp of random bases constituting the replicate barcode (BC) as pictured in **S3 Fig.** and **Fig. 4A**. This KanMX fragment was directionally cloned into Aat2-BamHI site of a pUC19 derivative with ScCEN5 cloned into the HindIII site KlURA3 cloned into the XbaI site creating the CFVs pAS006 (wlcr pool) and pAS007 (wrcl pool). In order to maintain a high complexity of the 12bp replicate BC, ~20,000-30,000 *Escherichia coli* colonies transformed with pAS006 and pAS007 respectively were scraped and used to prepare plasmid DNA (Wizard Miniprep) for yeast transformation.

The wlcr and wrcl yeast heterozygous deletion pools were each transformed with their appropriate CFV according to the following protocol. Two 2ml glycerol stocks of each pool were thawed from -80°C and used to inoculate 160ml of YPD+G418 (200ug/ml)+0.18ug/ml His. This culture was grown to mid-log phase (~7hrs at 30°C) cells were harvested and transformed with a modified Li-Ac protocol using ~400ng pABS006 or pABS007 linearized with SnaBI. Transformant colonies were plated to to C-Ura and allowed to grow for three days at 30°C. A total of 120,000-130,000 colonies were scraped for each pool. However, the transformation efficiency with CFVs pABS006 and pABS007 was only about 20% (as determined by a PCR assay, data not shown) so our pool of scraped colonies included both Tamp strains and original heterozygous deletion strains. However, as described below, the design of the PCR primers used to generate our Barseq sequencing libraries only amplified the strain-identifying barcode from Tamp strains and not from the original heterozygous deletion collection strains. Given this transformation efficiency, the total number of unique transformants collected was ~ 23,000 and ~20,000 for the wlcr and wrcl pools, respectively, resulting in ~18-21 unique replicates for each Tamp. Given the large number of replicate BCs included in the CFVs pABS006 and pABS007, each transformant was identifiable by a unique combination of the strain-identifying, as derived from the yeast deletion collection barcode (Tamp BC), and the replicate BC. The final wlcr and wrcl pools were each mixed at 1:1 ratio with 50% glycerol and 2ml aliquots were stored at -80°C.

To confirm the construction of this pool, we thawed an aliquot from each pool, extracted DNA with a modified Hoffman-Winston prep and prepared barseq libraries for sequencing using primers OAS021 to OAS023. These barseq libraries were prepared as described for the chrII-targeted Tamp pool and sequenced on an Illumina MiSeq with sequencing primers OAS024 to OAS027 generating 4,348,080 reads. The fastq files for this barseq experiment are at BioProject ID PRJNA257895 with BioSample IDs SAMN02979480 to SAMN029794821. Analysis of the barcodes sequenced in this run with a custom pipeline previously described [8] and custom python and R scripts revealed that on average each Tamp was represented by 105 (standard deviation = 135) replicate BCs and confirmed Tamp formation from 1802/2,254 target gene deletion strains. This confirmed that our pool was sufficiently complex to warrant further pooled competition experiments.

As revealed in the construction of our chrII-targeted Tamp pool, generating Tamps using CFVs was not an error-free process and variable karyotypes were sometimes produced. Unfortunately, this problem was exacerbated in the construction of the genome-wide Tamp pool with larger Tamps being more likely to have incorrect karyotypes. The most commonly observed incorrect karyotype was one where the Tamp initiated at the correct genomic location but did not extend all the way to the proximal telomere; this problem was most common for larger Tamps (**S7** **Table**). We adjusted our analysis pipeline to try and correct for these variable karyotypes.

Pooled competition experiments, barseq and fitness analysis of genome-wide Tamp pool

Similar to the chrII-targeted Tamp competition experiments, we inoculated nine total large volume (~300ml) nutrient-limited chemostats supplemented with 20mg/L histidine with aliquots of our wlcr and wrcl pools (both pools were inoculated into a single chemostat). We performed pooled competition experiments under the three different nutrient limited conditions (phosphate-, glucose- and sulfate-limited) in triplicate; chemostat inoculation and growth were the same as described for the chrII-targeted Tamp pool competition experiments. We defined each of the triplicate chemostat competition experiments as a technical replicate. For each of the nine chemostats, 10 timepoints were taken throughout the competition experiment. For each timepoint, DNA was extracted and two Barseq PCR reactions were carried out (one targeting wlcr Tamps and one targeting wrcl Tamps) using primers OAS021 to OAS023 and resulting in a total of 180 barseq samples. These 180 samples were pooled in equal proportions in two pools of 90 samples each. The pool, 6bp barcodes used for multiplexing and generations corresponding to each of the 180 samples are recorded in **S15 Table.** Each pool was sequenced on three lanes of an Illumina HiSeq generating a total of 752,336,013 reads. These fastq files are deposited at BioProject ID PRJNA257895 with BioSample IDs SAMN02979482 to SAMN02980021.

The number of reads identifying each replicate for each Tamp was determined using the previously described pipeline [8] in combination with a set of custom python scripts. In total we determined the read counts for 122,402 (glucose-limited), 128,584 (phosphate-limited), and 105,000 (sulfate-limited) Tamp biological replicates across 10 timepoints. As described above we used these read counts to determine the fitness of each Tamp. First, we removed all replicates that were represented by 0 reads at timepoint t = 0; due to the high complexity of our pool and the limited sequencing coverage occasionally no reads identified a particular replicate at the initial timepoint but did identify it at later timepoints. Second, we added ‘1’ to all read counts to remove all 0-count timepoints from our analysis. Third, we determined the frequency of each replicate at each timepoint and then calculated the log_2_(frequency at time = t / frequency at time = 0) generating a total of nine log_2_ ratios for each Tamp biological replicate. We determined the frequencies for the wlcr and wrcl pool Tamp replicates separately to account for any variation in the barseq PCR efficiency between the two pools. Fourth, we plotted the log_2_ ratios for each Tamp replicate vs. generations; we only used data from generations > 5 (ie. after steady-state was achieved) to determine the relative fitness for each Tamp replicate (an example of these data are plotted in **S5 Fig.)** We did not exclude Tamp biological replicates that were only tracked in one or two of the three technical replicate experiments. Fifth, to determine the relative fitness for each Tamp biological replicate, we examined the relationship between the log_2_ frequency for all available technical replicates and number of generations by fitting a linear model. More specifically, treating log_2_ frequency as the response and number of generations as the predictor, we fit both a linear regression model, and a piecewise linear regression model with one knot at the median number of generations. In both models, we allowed a separate intercept term corresponding to each of the three technical replicate chemostat experiments. To choose between these two models, we used the F-statistic for an ANOVA. If the F-statistic for an ANOVA selected the linear model, then we defined the relative fitness for that Tamp biological replicate as the slope of the linear regression. If piecewise linear regression was chosen, then we defined the relative fitness for that Tamp biological replicate to be the slope of the first piecewise linear function. This analysis was repeated for all biological replicates for a single Tamp, yielding a number of estimates of the relative fitness for that Tamp. Sixth, we combined the fitness estimates from all biological replicates into a single overall estimate of relative fitness for that Tamp, as follows. If there were three or fewer biological replicates for a given Tamp it was excluded from further analysis. If there were fewer than 15 biological replicates for a given Tamp, then the relative fitness and its associated standard error for that Tamp were computed as the mean and standard error of the relative fitnesses of all biological replicates, respectively. If a Tamp had more than 15 biological replicates, we defined relative fitness for that Tamp as the mode of the histogram of relative fitnesses of the biological replicates. The associated standard error was calculated using the bootstrap. We hoped that using the mode of the distribution of the biological replicate fitnesses to estimate the fitness of a given Tamp would reduce the error in this fitness estimate due to the incorrect karyotypes.

To further filter Tamps with high frequencies of incorrect karyotypes we removed from subsequent analyses all Tamps with errors greater than one standard deviation above the mean error for all Tamps. We reasoned that Tamps prone to karyotype variability would be more likely to produce biological replicates with large differences in fitness and thus could be filtered out by their larger error. The relative fitnesses for each Tamp under each condition were then normalized to the mean fitness of the pool under that particular condition. After completing these analyses we were able to calculate the relative fitness for 1,631, 1,596 and 1,551 different Tamps under glucose-, phosphate- and sulfate-limiting conditions respectively. Specifically, for the pooled Tamp competition carried out in sulfate-limiting conditions we obtained sequencing data sufficient to examine a total of 1,960 Tamps. 1,801 Tamps had more than 3 biological replicates and of these 1,551 had a standard error (SE) of their fitness estimate less than the mean standard error of all 1,801 Tamps plus one standard deviation (ie. SE < 0.076). Of the 1,551 Tamps included in our final analysis, 793 had 15 or fewer biological replicates and their fitnesses were estimated by the mean of the biological replicates. 758 had more than 15 biological replicates and their fitnesses were estimated by the mode of the distribution of the biological replicates. For the pooled Tamp competition carried out in glucose-limiting conditions we obtained sequencing data sufficient to examine a total of 1,985 Tamps. 1,849 Tamps had more than 3 biological replicates and of these 1,631 had a standard error of their fitness estimate less than the mean standard error of all 1,849 Tamps plus one standard deviation (ie. SE < 0.057). Of the 1,631 Tamps included in our final analysis, 704 had 15 or fewer biological replicates and their fitnesses were estimated by the mean of the biological replicates. 927 had more than 15 biological replicates and their fitnesses were estimated by the mode of the distribution of the biological replicates. For the pooled Tamp competition carried out in phosphate-limiting conditions we obtained sequencing data sufficient to examine a total of 1,977 Tamps. 1,850 Tamps had more than 3 biological replicates and of these 1,596 had a standard error of their fitness estimate less than the mean standard error of all 1,850 Tamps plus one standard deviation (ie. SE < 0.062). Of the 1596 Tamps included in our final analysis, 656 had 15 or fewer biological replicates and their fitnesses were estimated by the mean of the biological replicates. 940 had more than 15 biological replicates and their fitnesses were estimated by the mode of the distribution of the biological replicates. The relative fitness for each Tamp and the error, calculated as described above, are plotted for each condition in **S7 Fig**.

When we plotted the fitnesses for each Tamp across the genome, we observed that parts of the fitness profile had a stair step appearance where fitness plateaus were bordered by sharp fitness breakpoints. In order to segment the genome into regions defined by Tamps of similar fitness we applied the copy-number variant prediction software, DNAcopy [12], to our genome-wide fitness data using the following settings: we required a minimum of two adjacent fitness data to define a fitness plateau and a significance of 0.05 to call a fitness breakpoint. This segmentation defined a total of 250 fitness segments across the three different nutrient-limiting conditions (Colored boxes in **S7 Fig.**)

Comparing Tamp fitness data to single-gene amplification fitness data

Previously, our lab determined the fitness effects of single-gene amplifications genome-wide using pooled competition experiments followed by barseq of genome-wide ORF collections on both low-copy-number (CEN) high-copy-number (2μ) plasmids [8]. We compared these single-gene amplification data to our genome-wide Tamp data in two ways. First, we compared the kernel density estimates for the fitnesses defined by Tamps to the fitnesses defined by single-gene amplifications (**Fig. 7A**)**.** The kernel density estimates were computed in R. Second, we compared the pleiotropy of Tamps to single-gene amplifications by defining pleiotropy as the between-condition variance in fitness (**Fig. 7B**). We restricted our analysis to those strains that had three valid fitness estimates (i.e. one each from sulfate-, glucose- and phosphate-limiting conditions) sufficient to calculate a variance. The kernel density estimates for each distribution of variances was computed in R. Next, we stratified the 250 fitness segments as defined by DNAcopy as positive or negative and averaged the fitnesses of all single-gene amplifications contained within its length as determined by their low-copy-number (CEN) fitness effects (**S2D Fig.**) Finally, we examined the breaks between fitness plateaus as defined by our DNAcopy segmentation analysis and categorized each break as either an Upstep (ie. an increase in fitness moving along the chromosome towards the telomere) or a Downstep (ie. a decrease in fitness moving along the chromosome towards the telomere). We averaged the fitnesses, as determined by their low-copy-number fitness effects, of all single-gene amplifications contained within each breakpoint region plus one gene telomeric of the telomeric border of the breakpoint region (**Fig. 7C, S11 Table**). This extra gene was included simply to compensate for any insensitivity in the DNAcopy segmentation of our fitness data.

Identification of candidate driver genes by comparing Downstep genes with previously published datasets

As described in the main text we filtered the list of Downstep genes by comparing it to several published datasets: the list of genes commonly upregulated in clones evolved under glucose-, phosphate- or sulfate-limiting conditions [1,13], the list of genes that increased fitness when present on a low-copy number plasmid under glucose-, phosphate- or sulfate-limiting conditions [8], and the list of genes mutated in populations evolved under glucose-, phosphate- or sulfate-limiting conditions [8]. Specifically, for the comparison with the Payen, 2014 low-copy-number plasmid fitness data, we compared Downstep genes to Payen et. al.’s list of outlier fitness genes with fitnesses < -0.10 or > 0.10 (denoted as ‘CEN outlier’ in **S10** **Table** and **S12 Table**) and also to the set of genes with fitnesses greater than two standard deviations more than the mean fitness of that dataset (denoted as ‘CEN mean + 2SD’ in **S10** **Table** and **S12 Table**). CEN mean + 2SD genes still have extreme fitnesses but did not reach the stringent cutoff imposed in Payen, 2014 to be called ‘outliers’. For phosphate-limitation this included single-gene amplifications with fitnesses < -0.096 or > 0.097 and for glucose-limitation this included single-gene amplifications with fitnesses < -0.052 or > 0.050. The list of ‘outliers’ called by Payen *et al.* already included all mean + 2SD genes for sulfate-limited conditions.

**References:**

1. Gresham D, Desai MM, Tucker CM, Jenq HT, Pai DA, et al. (2008) The repertoire and dynamics of evolutionary adaptations to controlled nutrient-limited environments in yeast. PLoS Genet 4: e1000303. doi:10.1371/journal.pgen.1000303.

2. Miller AW, Befort C, Kerr EO, Dunham MJ (2013) Design and use of multiplexed chemostat arrays. JoVE: e50262. doi:10.3791/50262.

3. Vollrath D, Davis R, Connelly C, Hieter P (1988) Physical mapping of large DNA by chromosome fragmentation. Proceedings of the National Academy of Sciences of the United States of America 85: 6027.

4. Li H, Durbin R (2009) Fast and accurate short read alignment with Burrows-Wheeler transform. Bioinformatics 25: 1754–1760. doi:10.1093/bioinformatics/btp324.

5. Li H, Handsaker B, Wysoker A, Fennell T, Ruan J, et al. (2009) The Sequence Alignment/Map format and SAMtools. Bioinformatics 25: 2078–2079. doi:10.1093/bioinformatics/btp352.

6. Pashkova N, Gakhar L, Winistorfer SC, Sunshine AB, Rich M, et al. (2013) The yeast Alix homolog Bro1 functions as a ubiquitin receptor for protein sorting into multivesicular endosomes. Developmental Cell 25: 520–533. doi:10.1016/j.devcel.2013.04.007.

7. Robinson JT, Thorvaldsdóttir H, Winckler W, Guttman M, Lander ES, et al. (2011) Integrative genomics viewer. Nature Biotechnology 29: 24–26. doi:10.1038/nbt.1754.

8. Payen C, Sunshine AB, Ong GT, Pogachar JL, Zhao W, et al. (2015) Empirical determinants of adaptive mutations in yeast experimental evolution. bioRxiv: 1–58 Preprint. Available: http://www.biorxiv.org/content/early/2015/01/21/014068. doi: http://dx.doi.org/10.1101/014068.

9. Ho CH, Magtanong L, Barker SL, Gresham D, Nishimura S, et al. (2009) A molecular barcoded yeast ORF library enables mode-of-action analysis of bioactive compounds. Nature Biotechnology 27: 369–377. doi:10.1038/nbt.1534.

10. Morrow DM, Connelly C, Hieter P (1997) “Break copy” duplication: a model for chromosome fragment formation in *Saccharomyces cerevisiae*. Genetics 147: 371–382.

11. Tong AH, Evangelista M, Parsons AB, Xu H, Bader GD, et al. (2001) Systematic genetic analysis with ordered arrays of yeast deletion mutants. Science 294: 2364–2368. doi:10.1126/science.1065810.

12. Venkatraman ES, Olshen AB (2007) A faster circular binary segmentation algorithm for the analysis of array CGH data. Bioinformatics 23: 657–663. doi:10.1093/bioinformatics/btl646.

13. Ferea TL, Botstein D, Brown PO, Rosenzweig RF (1999) Systematic changes in gene expression patterns following adaptive evolution in yeast. Proceedings of the National Academy of Sciences of the United States of America 96: 9721–9726.
